# Monolayers of Amino Acid-Synthesized Gold Nanoparticles as SERS Substrates for Trace Chemical Sensing

**DOI:** 10.1021/acs.langmuir.5c01335

**Published:** 2025-07-22

**Authors:** Aleksandra M. Figat, Malwina Liszewska, Bogusław Budner, Bartosz Bartosewicz, Małgorzata Norek, Bartłomiej J. Jankiewicz

**Affiliations:** † Institute of Optoelectronics, 69698 Military University of Technology, gen. Sylwestra Kaliskiego 2, Warsaw 00-908, Poland; ‡ Faculty of Advanced Technologies and Chemistry, Military University of Technology, gen. Sylwestra Kaliskiego 2, Warsaw 00-908, Poland

## Abstract

In recent years, extensive studies have been devoted to the applications of surface-enhanced Raman spectroscopy (SERS) in detecting and identifying trace amounts of various analytes. The crucial issue for SERS applications is the selection of reliable and reproducible substrates. Therefore, most studies were dedicated to their fabrication, characterization, and evaluation using selected test compounds. This article reports the results of comparative studies on the fabrication, characterization, and evaluation of SERS substrates in the form of monolayers of amino acid-synthesized gold nanoparticles (AuNPs) on silicon and glass platforms. The combination of AuNPs synthesized with five amino acids and four platforms with smooth and rough surfaces yielded 17 potential SERS substrates. SERS substrates and their components were characterized using microscopy and spectroscopy techniques. The SERS performance of fabricated substrates was evaluated using *p*-mercaptobenzoic acid and 1,2-bis­(4-pyridyl)­ethylene as test analytes. The studies showed that not all AuNPs synthesized with amino acids are suitable for fabricating efficient SERS-active substrates. The AuNPs’ surface chemistry influences the fabrication and performance of SERS substrates. Among the fabricated SERS-active substrates, the highest enhancement factors (EFs) were estimated for those made with l-serine-synthesized AuNPs.

## Graphical abstract

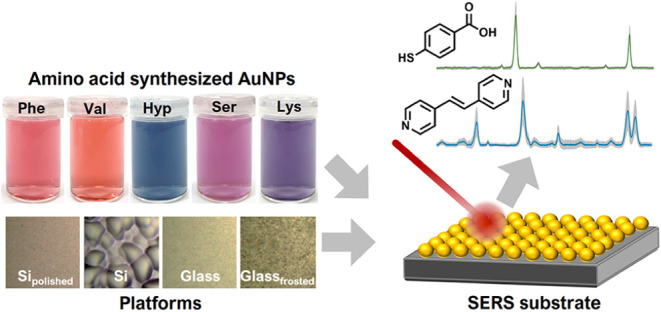

## Introduction

Surface-enhanced Raman spectroscopy (SERS) is a highly sensitive technique for detecting and identifying a wide range of analytes, which has been investigated for use in various applications.^1–3^ The potential practical applications of SERS span from medical diagnosis, biosensing, and bioanalysis^4–7^ to sustainable agriculture,^8^ food safety,^9^ and pharmaceutical analysis.^10^ SERS potential in defense and homeland security applications^11–13^ is particularly intriguing, as it can be used to detect and identify hazardous materials, such as chemical and biological agents, and explosive materials,^14–18^ to determine illicit drugs,^19^ and to analyze forensic evidence.^20^ Recently, we have also utilized SERS as a potential tool to study the reactions of reactive intermediates.^21^ The primary advantages of SERS over other currently used trace detection techniques include high sensitivity, rapid measurements, water compatibility, minimal sample preparation requirements, and the ability to measure both liquids and solids.^22^

Despite its potential, SERS remains primarily a laboratory technique. One of the critical issues hindering the practical application of SERS is the quality of the plasmonic substrates, which significantly influences the reliability of SERS measurements. SERS substrates dedicated to any application should be simple, reproducible, reliable, stable, ideally reusable, and cost-effective while providing homogeneous signal enhancement.^23^ In some cases, SERS substrates must also be tailored to specific applications.^24–27^ For example, SERS substrates are made flexible or in the form of swabs to collect the analyte directly from any rough surface by simple swabbing or swiping.^24,25^ Another example involves the functionalization of the SERS substrate surface with an affinity layer, such as antibodies, aptamers, or molecularly imprinted polymers, to achieve specificity toward target compounds and reduce background signals.^26,27^ The SERS substrates reported in the literature include various gold and silver nanostructures fabricated using chemical and physical methods.^24,28–32^ Due to the ease of fabrication, the most often studied SERS substrates are those based on metallic nanoparticles (NPs) and their assemblies.^31,32^ NPs-based SERS substrates come in various types based on how the NPs are structured, deposited, or assembled.^33–37^ They include solution-based substrates, such as colloidal NPs and their aggregates, and solid-based substrates, such as NPs monolayers, NPs-decorated surfaces, metallic nanostructured films, lithographically patterned NPs arrays, and metal NPs-based hybrid structures.^24,25,28–33^ The effectiveness of NPs-based SERS substrates depends on such factors as the NPs’ material,^38,39^ geometry (size and shape),^40–44^ arrangement on the SERS substrate platform, and the platform (i.e., the material and the type of platform).^24,25,45–48^

Depending on the type of NPs-based SERS substrate, NPs are fabricated using either chemical or physical methods.^1,32–37^ Specific physical methods have advantages over chemical methods, which include a lack of reagent contamination, good control over the shapes and sizes of metal nanostructures, the distance between them, and the ability to efficiently fabricate highly ordered SERS substrates.^49^ However, the main drawbacks of physical methods include limited access to often expensive instrumentation and fabrication costs associated with time-consuming processes involving multistep protocols. Despite some drawbacks, the chemical methods of fabricating SERS substrates continue to be intensively investigated.^1^ In the simplest case, gold (AuNPs) or silver (AgNPs) nanoparticles can be directly mixed with the analytes in a cuvette for SERS measurements.^50^ However, such measurements can be affected by the tendency of NPs to aggregate and the solubility of analytes. Hence, researchers have investigated various methodologies for assembling nanoparticles onto solid platforms (rigid or flexible) to enhance the performance of the resulting SERS substrates.^10,24,25^ In most cases, the fabrication of the NPs-based substrate is a multistage process involving the synthesis of nanoparticles and their assembly, often using a self-assembly approach, onto a solid platform. The surface of a solid platform can be chemically modified to introduce functional groups, improving the deposition of NPs and controlling their arrangement on the surface. The surface of assembled metal NPs can be functionalized to further enhance SERS performance in terms of sensitivity and selectivity toward analytes.^26,27,29^ The chemical methods for fabricating SERS substrates are cost-effective, enable the large-scale preparation of substrates, and do not require specialized instruments. Some methods enable the fabrication of SERS substrates with well-defined morphologies, resulting in uniform and reproducible SERS results that are sufficient for most applications.^29^

Herein, we report the results of comprehensive studies evaluating the suitability of amino acid-synthesized AuNPs for fabricating SERS substrates. As we have shown previously,^51^ α-amino acids are simple and readily available green reagents that can reduce gold ions and stabilize the synthesized AuNPs similarly to citrate ions used in the Turkevich method. Synthesis of AuNPs using standard Turkevich method conditions for most studied amino acids yielded spherical (mainly) or irregular particles with mean diameters from several to tens of nanometers and good stability. Therefore, we expected that, similarly to citrate-synthesized AuNPs, the AuNPs synthesized with amino acids would be suitable for the fabrication of SERS substrates.^52^ SERS substrates were fabricated by depositing five different types of amino acid-synthesized AuNPs, which vary in size (from ca. 20 to 70 nm for spherical NPs), shape (spherical, nanoclusters, and irregular), surface chemistry (different capping agents), and optical properties, on four amino-functionalized glass and silicon platforms, which vary in surface roughness. As a result, we obtained 17 potential SERS substrates, which were characterized using microscopy (TEM, SEM, AFM) and spectroscopy (UV–vis, XPS) techniques. Finally, we compare the performance of the fabricated SERS substrates in terms of their response to 4-mercaptobenzoic acid (pMBA) and 1,2-bis­(4-pyridyl)­ethylene (BPE) molecules adsorbed onto them.^53^ Thanks to the multivariate comparative analysis, it was possible to observe the significant role of the size and surface chemistry of metallic nanoparticles in obtaining high-performance SERS substrates. To our knowledge, no research has yet presented a similar comprehensive analysis of the influence of multiple combinations of different platforms and AuNPs of various shapes, sizes, and surface chemistry on SERS substrate performance.

## Results and Discussion

### Fabrication of SERS Substrates

In our research studies, we focus, among many, on developing simple fabrication methods for SERS substrates from easily accessible materials. In studies reported herein, all AuNPs-based SERS substrates were fabricated using the procedure shown in Figure 1. Five different amino acid-synthesized AuNPs were deposited on four functionalized glass and silicon platforms with varying surface roughness (Figures S1 and S2, Table S1), using the same fabrication protocol.

**Figure 1:**
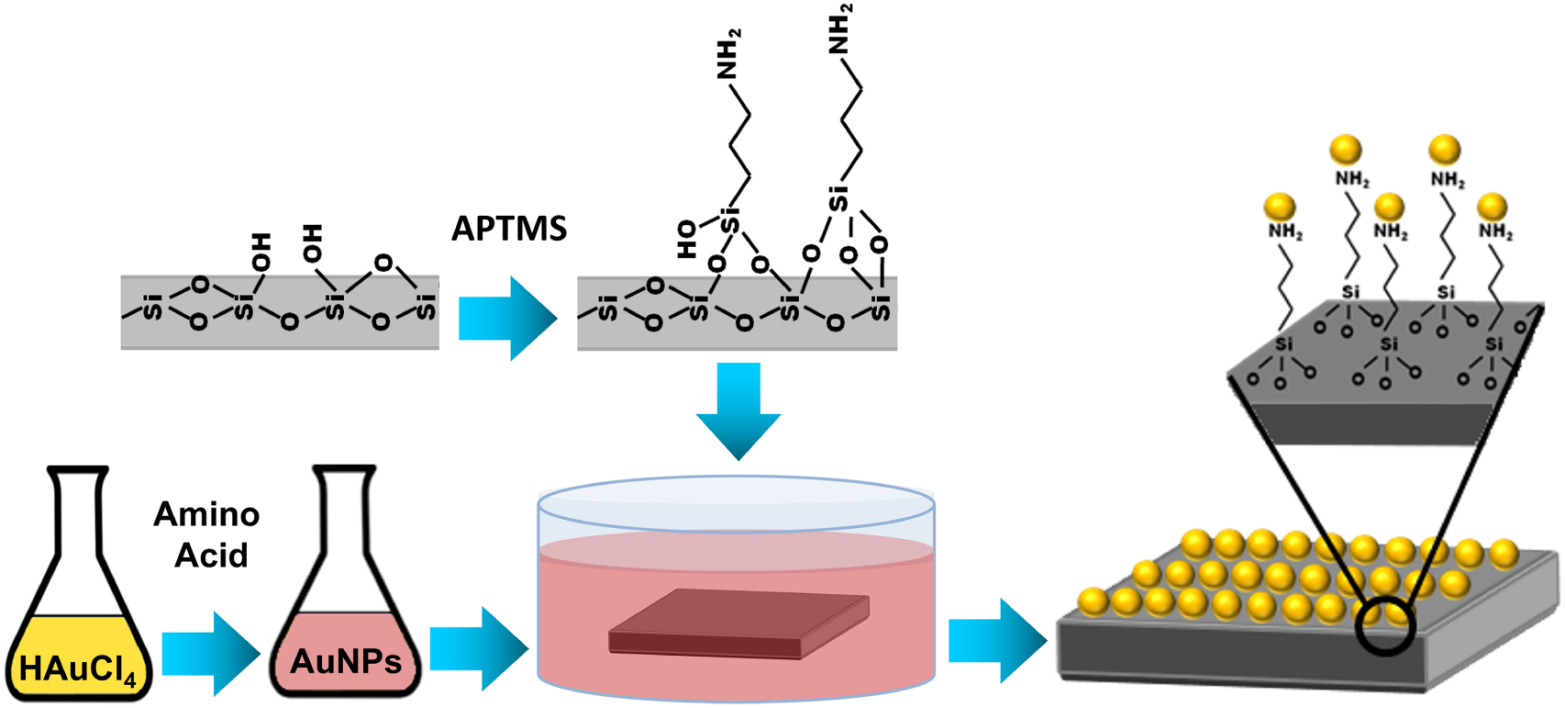
Overview of the fabrication process of AuNP-based SERS substrates.

The AuNPs of various shapes and/or sizes, as shown in Figures 2 and S3–S7, were synthesized using selected α-amino acids according to a method previously described by us.^51^ The selection of amino acids used in reported studies was based on a careful analysis of the obtained results,^51^ considering factors such as size, shape, surface chemistry, and optical properties. AuNPs synthesized with dl-phenylalanine (Phe), l-valine (Val), and l-serine (Ser) are representatives of 12 amino acid-synthesized AuNPs with a quasi-spherical shape. AuNPs synthesized with Val and Ser were monodisperse quasi-spherical NPs with mean diameters of ca. 30 and 70 nm, respectively. AuNPs synthesized with Phe were polydisperse quasi-spherical NPs with diameters ranging from ca. 15 to 25 nm. AuNPs synthesized using the two remaining α-amino acids, l-lysine (Lys) and l-(4)-hydroxyproline (Hyp), were irregular particles with sizes of 60–70 nm or nanoclusters with sizes of 25–95 nm, respectively. In each case, the α-amino acid served as both a reducing and capping agent.

**Figure 2:**
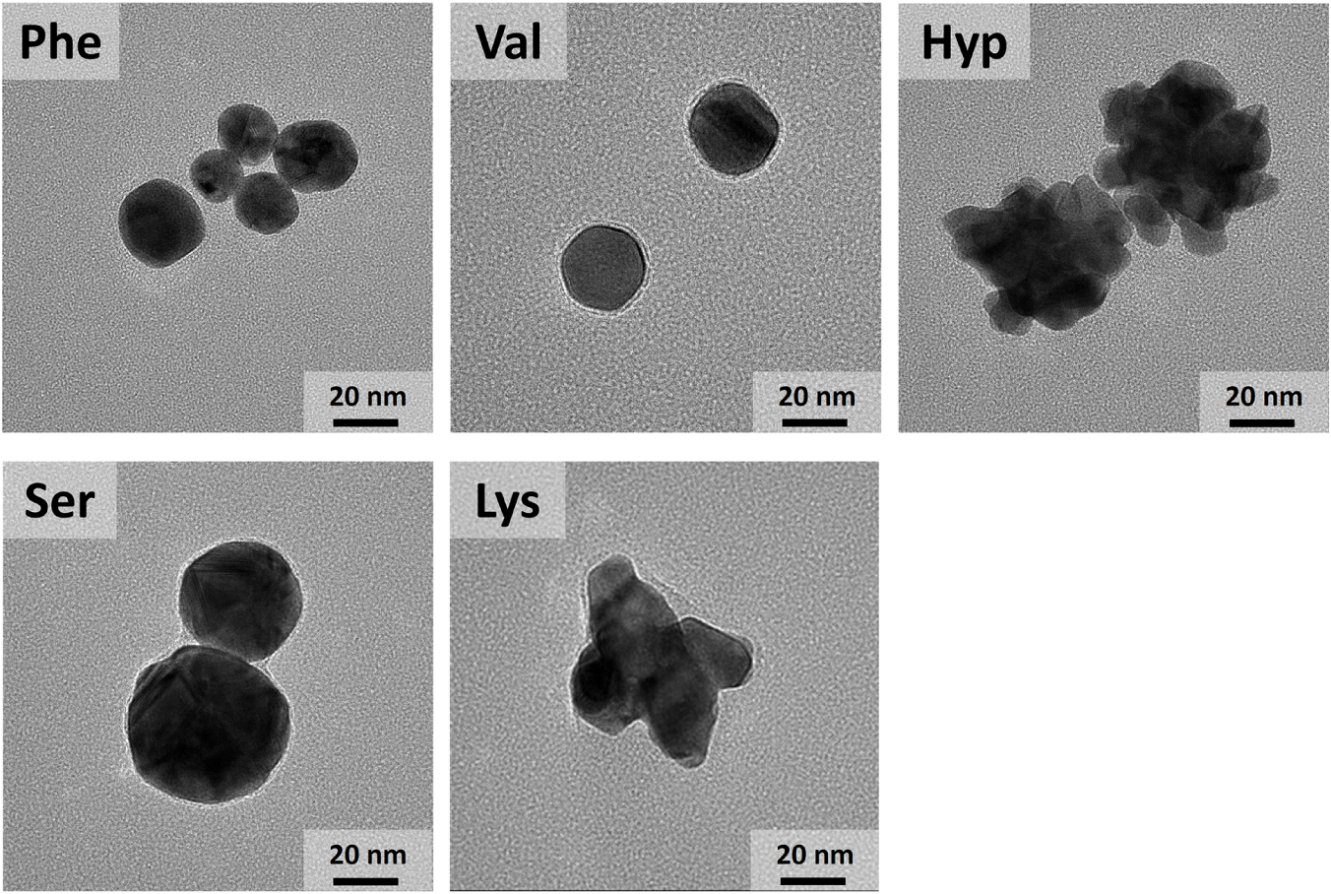
TEM images of gold nanoparticles used in these studies.

AuNPs were deposited on four amino-functionalized glass and silicon platforms with different surface roughnesses. Two types of silicon platforms represent the rough and smooth sides of a single-sided polished silicon wafer. Two types of glass platforms represent the smooth and specially frosted microscope slide. The differences in measured surface roughness values were much larger for glass than for silicon platforms. The increased surface roughness of the platform was expected to result in a higher number of introduced functional binding groups and, therefore, more densely packed AuNPs on the resulting SERS substrate.

The glass and silicon platforms were modified with (3-aminopropyl)­trimethoxysilane (APTMS) to introduce functional amino groups on the platforms’ surfaces, aiming to bind AuNPs via physical sorption (Figure 1). XPS measurements were conducted to investigate changes in the surface chemistry of the platforms and SERS substrates during the fabrication process. Based on the XPS spectra (Figures S8–S9), the smooth glass substrate has a nitrogen content of 0.24 at% after the cleaning procedure (cleaning in piranha solution and then washing with DI water and acetone), which increases to 1.74 at% after the functionalization process using aminosilanes. The nitrogen content of the frosted glass, which has a more developed surface, was 0.51 at% after the cleaning procedure and increased to 3.06 at% after functionalization. The differences in N content are related to the quantity of amino-functional groups attached to the glass surface during functionalization, which is higher in the case of frosted glass. After cleaning, the silicon platforms had nitrogen contents of 1.09 at% and 0.13 at% for polished and rough surfaces, respectively. These contents increased after functionalization to 1.73 at% and 1.88 at%, respectively. A summary of the XPS test results for glass and silicon platforms after the deposition of AuNPs on functionalized platforms is presented in Table S2. The chemical composition analysis of the substrates’ surface reveals the presence of O, N, C, Si, and Au, with the C source being organic, derived from the synthesis of AuNPs and APTMS surface functionalization. In the case of SERS substrates based on Si platforms, the general trend of element content is similar for both polished and rough platforms when AuNPs are synthesized with the same reducing agent. The carbon content is the highest for the SERS substrates made of AuNPs (between 43.1 at% and 45.1 at%) synthesized with Phe, the amino acid with the highest number of C atoms in its structure compared to other amino acids. The increased carbon content can be attributed to the amino acid-stabilizing layer surrounding AuNPs. The amount of Au is on a similar level for the SERS substrate made of AuNPs synthesized with Val and Ser, and is, respectively, 7.7 at% (Table S2 and Figure S10) for both samples on Si_polished_ and between 8.1 at% and 8.2 at% for samples on rough Si. In the case of SERS substrates made by deposition of AuNPs of the same size on different Si platforms, the differences in Au content suggest that there are more AuNPs on a rough than a polished Si platform. The Au content is also higher on SERS substrates made on frosted glass platforms compared to those made on smooth glass platforms. The Au content was lowest for all SERS substrates fabricated with Hyp-synthesized AuNPs. The XPS measurement results are consistent with the observations based on SEM images.

The contact angle measurement revealed that all platforms and SERS substrates were hydrophilic, with contact angles less than 90° (Table S3). The contact angle of platforms increased after functionalization with aminosilanes. Contact angle behavior showed no clear trend following the deposition of different types of amino acid–synthesized AuNPs on the functionalized platforms, likely due to a specific interplay between the surface chemistry of the AuNPs, which varied depending on the amino acid used for their synthesis, and their distribution on the platform. As shown in Table S3, for SERS substrates made on Glass_frosted_, Si_polished_, and Si platforms with Phe, Val, and Ser-synthesized AuNPs, we observed an increase in contact angle compared to functionalized platforms. Interestingly, we observed a decrease in contact angle for SERS substrates made with the mentioned AuNPs on the Glass platform. Contact angles for SERS substrates made with Hyp-synthesized AuNPs either remain the same (Glass platform) or decrease. The only SERS substrate that we were able to fabricate using Lys-synthesized AuNPs had a lower contact angle compared to the functionalized Glass_frosted_ platform.

In the final step of fabricating SERS substrates, AuNPs were deposited from the solution onto functionalized platforms (Figure 1). The deposition process is complex and can be influenced by various factors, including the concentration of the colloidal solution, pH, ionic strength, particle size, capping agents, platform surface chemistry, and immersion time.^54^ Our study focused on the AuNPs used (size, shape, and capping agent); therefore, the platform surface chemistry and deposition conditions (i.e., immersion time) were kept constant throughout the experiments, as reported in the experimental section. We also used colloidal solutions as obtained in the synthesis.

SEM images of all fabricated SERS substrates are shown in Figures 3 and S11–S14. Only 17 of the expected 20 potential SERS substrates were obtained because AuNPs synthesized with l-lysine deposited only on Glass_frosted_ and not on the other platforms used, as shown in Figure S14. The morphology of fabricated substrates is influenced by the components they are made of, the platforms and the AuNPs used. The platforms’ surface coverage with AuNPs depends on their surface roughness, as reported in the literature (Table S1).^55^ This observation is especially evident in the case of glass platforms, where differences in surface roughness are significant, resulting in more densely packed AuNPs on frosted glass. This fact is confirmed by the increased absorptance of the SERS substrates on frosted glass, as shown in Figure 4. In the case of Si platforms, there is no visible difference in surface coverage with AuNPs between smooth and rough platforms due to their similar surface roughness (Figures 3 and S11–S13). However, XPS results indicate that slightly more AuNPs are deposited on rough Si platforms. Interestingly, AuNPs are more densely packed on polished Si than on smooth glass platforms.

**Figure 3:**
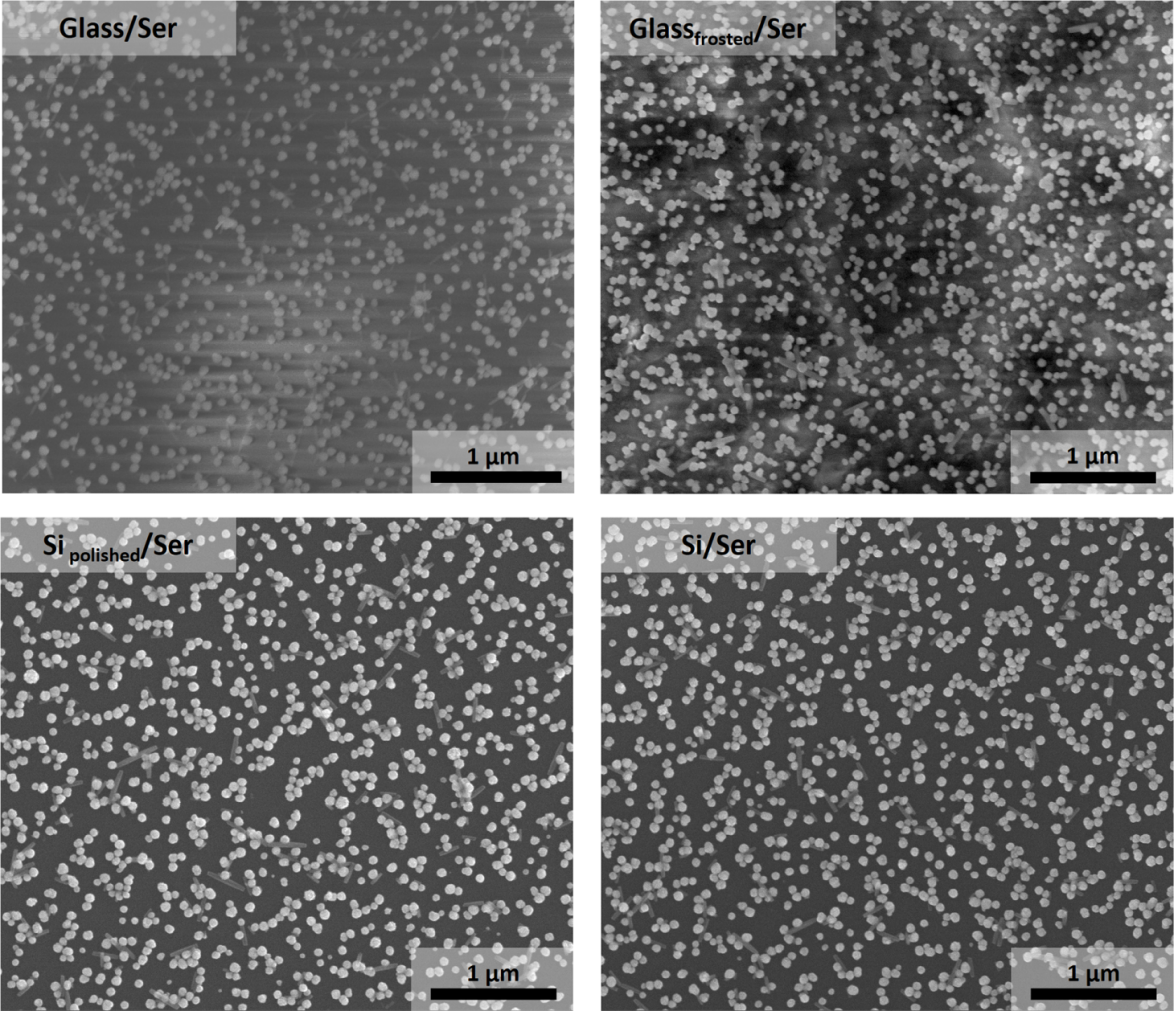
SEM images of SERS substrates fabricated using AuNPs synthesized with l-serine. SERS substrates were made using the following platforms: glass (upper left), frosted glass (upper right), polished silicon (bottom left), and silicon (bottom right).

**Figure 4:**
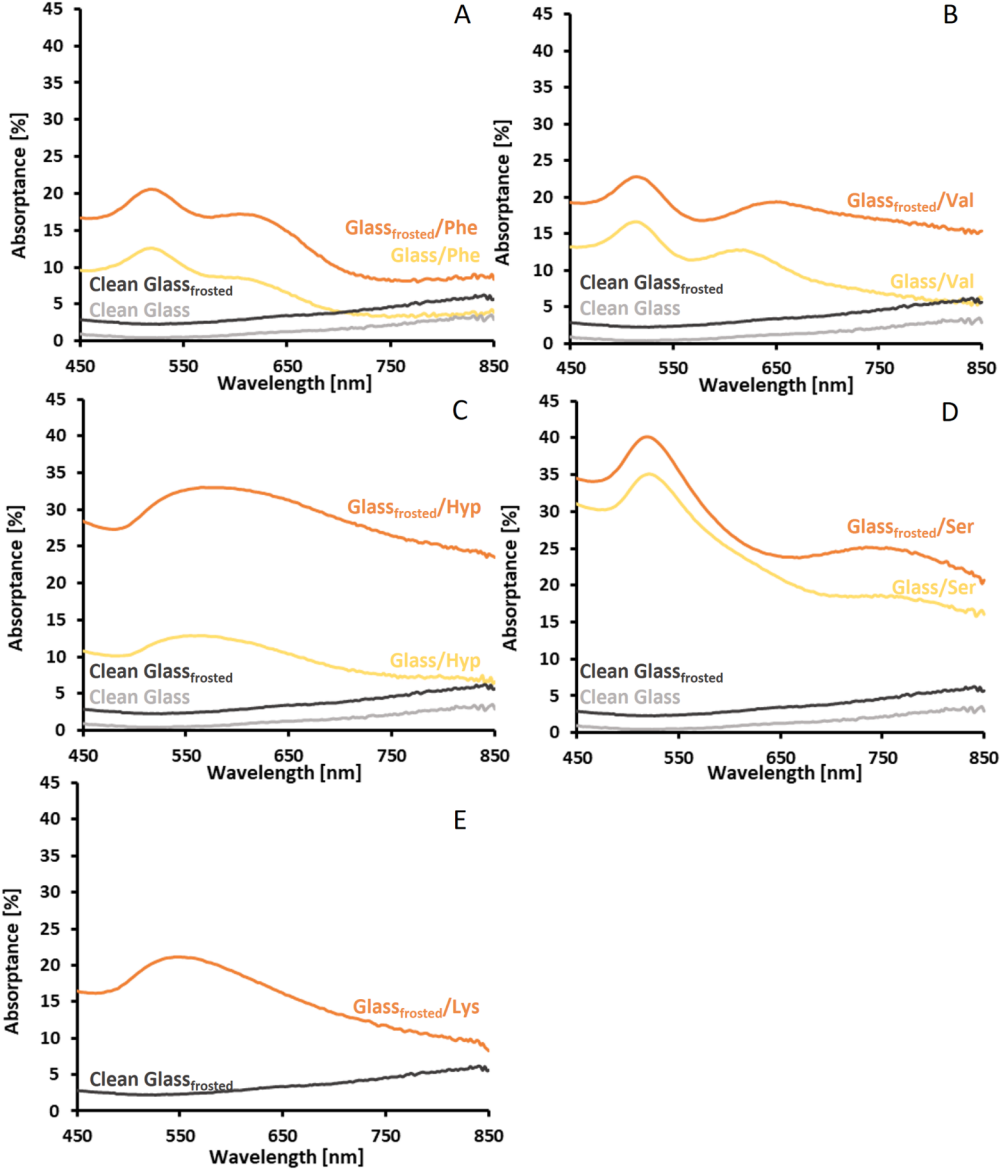
Absorptance spectra of glass platforms (smooth and frosted) and corresponding SERS substrates fabricated using AuNPs synthesized with dl-phenylalanine (A), l-valine (B), l-(4)-hydroxyproline (C), l-serine (D), and l-lysine (E).

The deposition of AuNPs is also related to their surface chemistry and the strength of interaction between amino acids and the surface of AuNPs.^54,56–58^ In fact, AuNPs’ surface chemistry, and not the surface contact area corresponding to AuNPs’ size, has been previously shown to be the dominating factor for AuNPs’ self-organization.^54^ SERS substrates fabricated with AuNPs synthesized with Phe, Val, and Ser showed good surface coverage. These observations are likely associated with medium-strength to weak interactions between these amino acids and the surfaces of AuNPs, in comparison to other amino acids,^56–58^ which allows for good binding to the amine groups on the platform’s surface. Based on molecular dynamics simulations of solvated amino acids reported elsewhere,^56^ all of the amino acids prefer to contact the gold surface with at least part of their backbone. In addition, the microscopic effects (the competition between the gold-affinity, solvation propensity, and the geometric preferences of the various chemical groups composing each amino acid) tend to favor orientations where the charges are exposed to the solvent.^56^ For example, the reported results of simulations for Phe^56^ showed that the side chain of Phe is only partially in contact with the surface, featuring a conformation with a tilted aromatic ring and optimal backbone contact. These results are in good agreement with experimental SERS studies,^59^ in which, based on the analysis of SERS spectra, it was assumed that the orientation of Phe molecules on the surface of AuNPs closely resembles the parallel orientation.

Interesting observations were made for substrates fabricated with AuNPs synthesized with Hyp. These AuNPs were deposited on all the used platforms; however, the fabricated substrates were shown to be unsuitable for SERS, as discussed later. The Hyp, similarly to l-proline,^57,58^ is expected to have medium strength interactions with the surface of AuNPs when compared to other amino acids. These interactions have not been studied previously, but Hyp molecules should interact with the AuNPs surface similarly to l-proline through the pyrrolidine ring.^56^ Similar to other used amino acids, the expected strength of the interactions with the Au surface allows Hyp-synthesized AuNPs to bind well to all platforms. However, such substrates exhibit a rich background spectrum that is undesirable for SERS substrates. A possible explanation was provided by carefully examining the SERS and TEM results (Figures 2 and S5). Hyp-synthesized AuNPs were the only AuNPs synthesized with amino acids, which had a form of nanoclusters.^51^ As seen on TEM images and EDX spectra (Figure S5), these nanoclusters consist of multiple smaller Au nanoparticles bound together and have a significant amount of C and O. As mentioned by us previously,^51^ the mechanism of gold salts reduction by Hyp was expected to be different compared to other studied amino acids. Based on the results of the studies reported herein, the stabilization of AuNPs formed during synthesis with Hyp can also occur differently. During the synthesis of AuNPs, Hyp molecules, in addition to other reactions, can also undergo peptidization. This process has been previously demonstrated to occur for Hyp in an aqueous solution, even in the absence of a catalyst.^60^ The oligomers formed during the Hyp peptidization can bind small Au nanoparticles to form final Au nanoclusters, which Hyp-oligomers also stabilize. These would explain the higher amounts of C and O in the EDX analysis (Figure S5). The Hyp-synthesized AuNPs bind to the platforms’ surface through interactions of the Hyp-oligomers’ functional groups (e.g., COOH) with amine groups on the platforms’ surface. At the same time, the Hyp-oligomers form a protective shell, limiting the adsorption of Raman reporters’ molecules on the AuNPs surface and/or separating them from the “hot spots”, resulting in a rich background spectrum and low-intensity SERS signals of reporters.

The AuNPs fabricated and capped with l-lysine deposited only on the surface of the frosted glass. A possible explanation for this observation is the extraordinary colloidal stability of Lys-capped AuNPs caused by the strong binding of Lys to AuNPs.^61^ Lys molecules bound strongly to the AuNPs’ surface via the amino group attached to the α-carbon, and carboxylic and ε-amino groups of Lys extended out to stabilize the AuNPs.^56–58,61,62^ These relatively strong binding of Lys molecules to the AuNPs surface in comparison to other amino acids,^57,58^ the ε-amino groups of Lys side chain extended out to the solvent, and the relatively large size of the AuNPs may prevent their deposition on platforms with a low number of amino-functional groups available for binding. In the case of frosted glass, where many functional groups are available, Lys-capped AuNPs bind to the platform’s surface.

### The Optical Properties of SERS Substrates

The absorptance spectra of SERS substrates fabricated on glass and Si platforms are shown in Figures 4 and 5. The deposition of amino acid-synthesized AuNPs on functionalized platforms yields SERS substrates whose optical properties differ from those of suspensions of AuNPs, which they are made of.^51^ These observations can be explained by the shortening of interparticle spacings on the SERS substrates compared to AuNP sols. This leads to stronger electromagnetic interactions between AuNPs through a dipole coupling mechanism.^63^ All SERS substrates investigated in this work absorb light in the visible range and exhibit characteristic plasmon resonance maxima ranging from ca. 510 nm to ca. 600 nm.^63^ Comparing the light absorption level for SERS substrates made on glass platforms reveals that an increase in surface roughness increases the number of deposited AuNPs, resulting in increased light absorption. A shift in absorption maxima and broadening of absorption bands compared to AuNPs sols are observed for all substrates. Additional absorption bands in the spectral range above 600 nm are observed for substrates fabricated using AuNPs synthesized with Phe, Val, and Ser. These absorption bands correspond to the light absorption by nanoparticles agglomerated on the substrates’ surface. The differences in light absorption levels of SERS substrates made on smooth and rough Si platforms are almost negligible. This observation is associated with very similar surface roughness and, thus, similar surface coverage with AuNPs. The shift in absorption maxima and broadening of absorption bands are observed for all substrates made on Si platforms. The absorption bands with maxima at approximately 750 nm observed for SERS substrates made using AuNPs synthesized with Phe likely result from the formation of AuNPs’ chains consisting of 2–5 nanoparticles (Figure S11). Such structures follow a concept of plasmonic polymer, whose collective optical properties depend on the repeat unit.^64^ The optical properties of these SERS substrates are the sum of the optical properties of single nanoparticles, chains, and aggregates of Phe-synthesized AuNPs.

**Figure 5:**
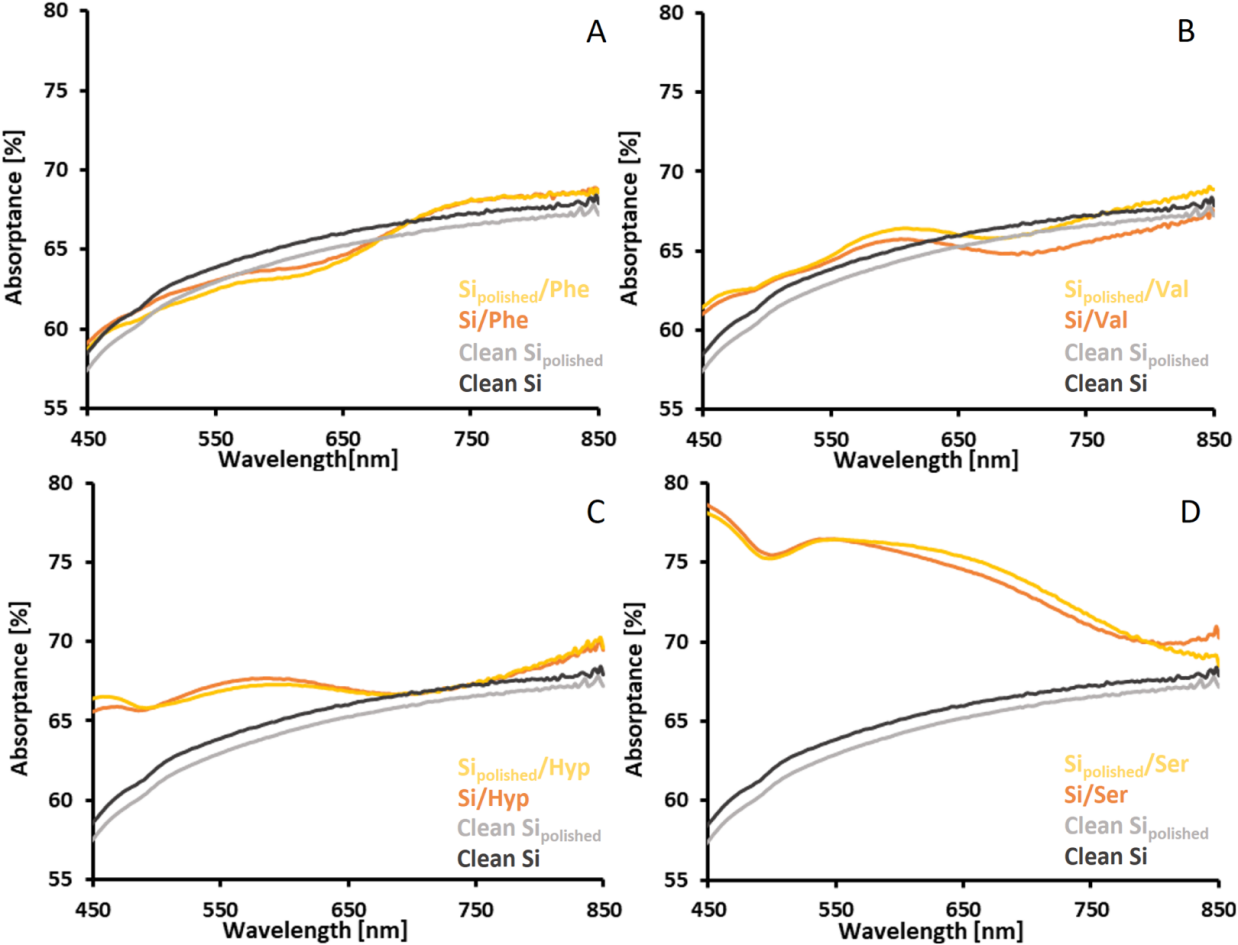
Absorptance spectra of Si platforms (smooth and rough) and corresponding SERS substrates fabricated using AuNPs synthesized with dl-phenylalanine (A), l-valine (B), l-(4)-hydroxyproline (C), and l-serine (D).

### SERS Performance of Fabricated SERS Substrates

The SERS performance of the fabricated substrates was evaluated using two Raman reporters commonly used to quantify the SERS enhancement factor (EF), p-mercaptobenzoic acid (pMBA) and 1,3-bis­(4-pyridyl)­propane (BPE). The pMBA binds to the gold surface through the thiol group, whereas BPE binds via the nitrogen atom of the pyridyl ring.^65^ Importantly for EF estimation, pMBA can form self-assembled monolayers (SAMs) with a known packing density on Au surfaces.^66^ The SERS EFs for pMBA and BPE were estimated using eq 1:

1 $$\mathrm{EF}=\frac{I_{\mathrm{SERS}}/N_{\mathrm{surf}}}{I_{\mathrm{RS}}/N_{\mathrm{vol}}}$$

by comparing the ratios of the SERS peak intensity (I_SERS_) of the reporter monolayer on the SERS substrate to the intensities (I_RS_) of the corresponding unenhanced signals from neat reporter films of known thickness.^67^ N_surf_ and N_vol_ correspond to the number of reporter molecules adsorbed on a SERS substrate surface and in a neat reporter film, respectively.^67^ Additional information regarding SERS EF estimation is provided in the Supporting Information.

The statistical analysis of the results obtained included determining the average intensity of the most intense vibrational modes at 1075 cm^–1^ (attributed to aromatic ring breathing, symmetric C–H in-plane bending and C–S stretching^68^) for pMBA and at 1200 cm^–1^ (attributed to ethylenic C=C stretching^69^) for BPE, the standard deviation of the peak intensity, and the maximum intensity of this peak from registered maps. Evaluating these parameters allowed us to determine which samples have the highest average EF of the Raman signal, the most uniform distribution of “hot spots,” and the maximum EF from the best “hot spot” on each substrate. Table 1 and Figure 6 summarize the statistical analysis results of the acquired SERS spectra of pMBA and BPE. The reducing and capping agents (amino acids) used to synthesize AuNPs are listed in order of the increasing size of nanoparticles made with their use.

**Figure 6:**
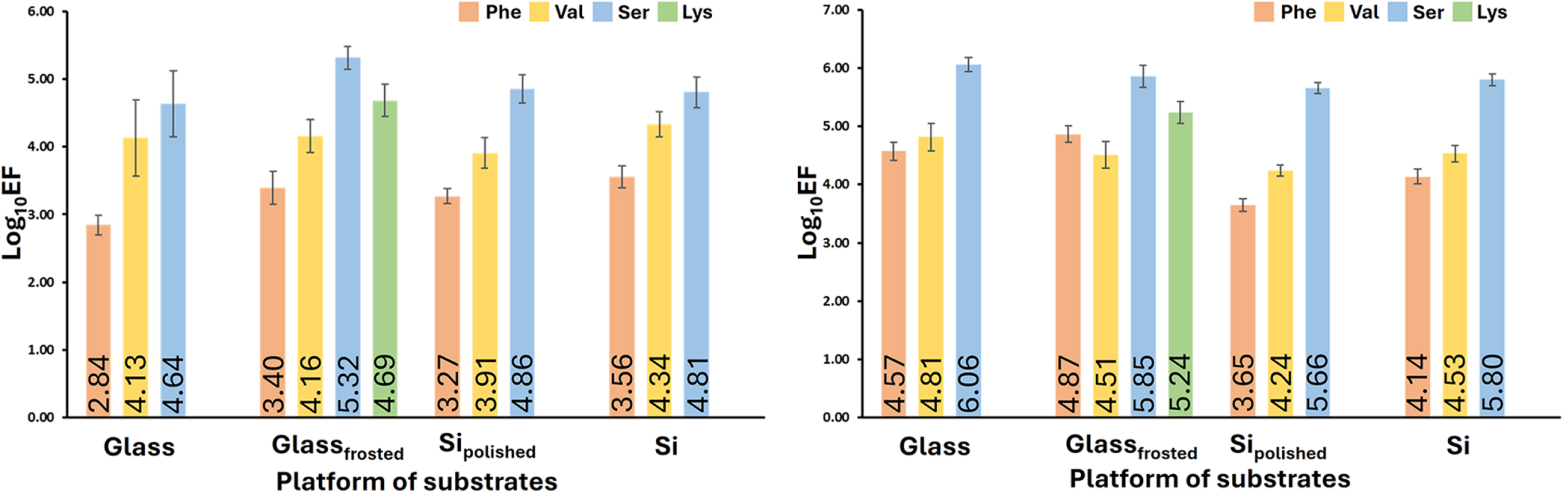
Graphs of log_10_ EF obtained based on the results of measurements with pMBA (left) and BPE (right).

**Table 1:** EFs Estimated for Measurements with pMBA and BPE Using Fabricated SERS Substrates^a^

	**Platform**	Glass			**Glass**_**frosted**_		
AuNPs made with	Raman Reporter	EF	SD	EF_max_	EF	SD	EF_max_
**Phe**	pMBA	6.99 × 10^2^	2.36 × 10^2^	1.45 × 10^3^	2.50 × 10^3^	1.41 × 10^3^	8.81 × 10^3^
	BPE	3.75 × 10^4^	1.35 × 10^4^	7.64 × 10^4^	7.35 × 10^4^	2.30 × 10^4^	1.61 × 10^5^
**Val**	pMBA	1.36 × 10^4^	1.77 × 10^4^	1.62 × 10^5^	1.46 × 10^4^	8.05 × 10^3^	4.46 × 10^4^
	BPE	6.51 × 10^4^	3.61 × 10^4^	2.30 × 10^5^	3.22 × 10^4^	1.71 × 10^4^	1.21 × 10^5^
**Hyp**	pMBA	SERS EF could not be reliably determined					
	BPE	SERS EF could not be reliably determined					
**Ser**	pMBA	4.33 × 10^4^	4.83 × 10^4^	2.46 × 10^5^	2.10 × 10^5^	8.15 × 10^4^	6.52 × 10^5^
	BPE	1.14 × 10^6^	3.15 × 10^5^	2.24 × 10^6^	7.11 × 10^5^	3.07 × 10^5^	2.84 × 10^6^
**Lys**	pMBA	No deposition of AuNPs			4.90 × 10^4^	2.65 × 10^4^	1.46 × 10^5^
	BPE	No deposition of AuNPs			1.72 × 10^5^	7.55 × 10^4^	4.06 × 10^5^

	**Platform**	Si_**polished**_			**Si**		
**AuNPs made with**	**Raman Reporter**	EF	SD	EF_max_	EF	SD	EF_max_
**Phe**	pMBA	1.87 × 10^3^	4.58 × 10^2^	3.88 × 10^3^	3.60 × 10^3^	1.34 × 10^3^	1.13 × 10^4^
	BPE	4.46 × 10^3^	1.11 × 10^3^	7.34 × 10^3^	1.38 × 10^4^	3.89 × 10^3^	2.34 × 10^4^
**Val**	pMBA	8.14 × 10^3^	4.30 × 10^3^	1.96 × 10^4^	2.18 × 10^4^	9.30 × 10^3^	6.08 × 10^4^
	BPE	1.73 × 10^4^	3.94 × 10^3^	2.70 × 10^4^	3.39 × 10^4^	1.11 × 10^4^	9.19 × 10^4^
**Hyp**	pMBA	SERS EF could not be reliably determined					
	BPE	SERS EF could not be reliably determined					
**Ser**	pMBA	7.30 × 10^4^	3.50 × 10^4^	1.73 × 10^5^	6.41 × 10^4^	3.40 × 10^4^	2.48 × 10^5^
	BPE	4.55 × 10^5^	9.83 × 10^4^	6.43 × 10^5^	6.26 × 10^5^	1.48 × 10^5^	9.25 × 10^5^
**Lys**	pMBA	No deposition of AuNPs					
	BPE	No deposition of AuNPs					

a EF—the average enhancement factor, SD—the standard deviation of the average enhancement factor, EF_max_—the maximum enhancement factor on each substrate

As mentioned, only 17 out of the expected 20 SERS substrates were obtained (Table 1 and Figure 6). All substrates fabricated with AuNPs synthesized with Val, Phe, Ser, and Hyp showed SERS activity. Lys-synthesized AuNPs deposited only on frosted glass, and the substrate obtained was SERS-active. In measurements with pMBA, the EF increases with the size of the deposited AuNPs (Table 1 and Figure 6). The EF ranges from 6.99 × 10^2^ to 3.60 × 10^3^ for the smallest nanoparticles made with Phe and increases to 2.10 × 10^5^ for the largest AuNPs synthesized with Ser. This effect can be attributed to the increased scattering efficiency of metallic nanoparticles as their size increases. The estimated EFs for the measurements with BPE follow the same trend as for pMBA, except for results obtained on SERS substrates made using frosted glass. The EF ranges from 4.46 × 10^3^ to 7.35 × 10^4^ for the smallest nanoparticles made with Phe and increases to 1.14 × 10^6^ for the largest AuNPs synthesized with Ser. For both Raman reporters, the highest EFs were estimated for all SERS substrates made with Ser-synthesized AuNPs. This observation is consistent with the results of previously reported studies, in which it has been shown that SERS enhancement increases with the increasing size of AuNPs.^70^ This observation was assumed to be associated with an increase in the electromagnetic enhancement. With the increasing size of the NPs and their aggregation, the corresponding localized surface plasmon resonances (LSPR) band maximum (λ_max_) shifts to longer wavelengths and broadens. Previous studies have shown that to achieve optimal SERS enhancement, the laser excitation line should be slightly blue-shifted with respect to the LSPR maximum, so that the λ_max_ is located between the excitation and the Raman lines.^71^ However, more recently, it has been shown that the optimal position is a compromise between the positions of the Raman and laser lines, compared to the LSPR, and the intensity of the near-field band.^72^ In our studies, it was found that the average EF values for pMBA are higher on SERS substrates made on roughened platforms. However, this is not the case for BPE measurements on frosted glass. For both Raman reporters, the EFs values trend is the same for SERS substrates made on smooth glass and polished and rough silicon (Table 1 and Figure 6). The only anomaly is observed in the results obtained for BPE measured on SERS substrates on frosted glass. For all SERS substrates, estimated EFs had higher values for BPE than for pMBA. The standard deviations of EF for BPE are much smaller than for pMBA. This observation is likely associated with the reporters’ molecules’ structure and their adsorption on the surface of the SERS substrate.

The pMBA and BPE spectra acquired for SERS substrates made with Ser-synthesized AuNPs are shown in Figure 7. The purple and green lines represent the average spectrum acquired using SERS substrates based on Si platforms with smooth and rough surfaces. The orange and blue lines represent the average spectrum acquired using SERS substrates based on glass platforms with smooth and frosted surfaces. The shaded area represents the standard deviation around the average spectrum. To better visualize the spectra of SERS substrates, the intensities of specific spectra were multiplied by the scaling factor depicted in the figures (e.g., x20 means multiplying the spectrum intensity by 20). Additionally, the 2D map of BPE SERS intensity at 1200 cm^–1^, acquired to show the distribution of “hot spots”, is shown in Figure 8. These SERS substrates have some additional peaks in their background spectra beyond those originating from glass or silicon. These may arise from l-serine molecules adsorbed on the AuNPs surface or residues from the synthesis. For the pMBA, the highest EF of 2.10 × 10^5^ was estimated for the SERS substrate fabricated on frosted glass, which is almost five times higher than that for the SERS substrate fabricated on smooth glass. The standard deviation of the EF for the SERS substrate on glass is higher than the average EF, indicating that substrates produced on glass platforms exhibit regions characterized by either very high or a lack of Raman signal enhancements. Therefore, these SERS substrates can be characterized by a low density of “hot spots” on their surfaces. Both Si platforms have similar EFs, which were 3× lower than the EF of the SERS substrate fabricated on frosted glass. SERS substrates on Si platforms have similar standard deviations and maximum EFs. For BPE, the highest EF of 1.14 × 10^6^ among SERS substrates made with Ser-synthesized AuNPs and all investigated SERS substrates was estimated for the SERS substrate fabricated on smooth glass (Table 1). In addition, the highest maximum EF of 2.84 × 10^6^ was obtained on SERS substrates fabricated by depositing Ser-synthesized AuNPs on frosted glass. As shown by the standard deviation of the EF, the well-enhancing “hot spots” are more evenly distributed on the surface of smooth glass, resulting in a higher average EF than in the case of the frosted glass platform, characterized by single “hot spots” with high enhancement (Table 1 and Figure 6). In the case of Si platforms, the rough Si platform enabled us to obtain an average EF almost 1.4 times higher than that of polished Si platforms.

**Figure 7:**
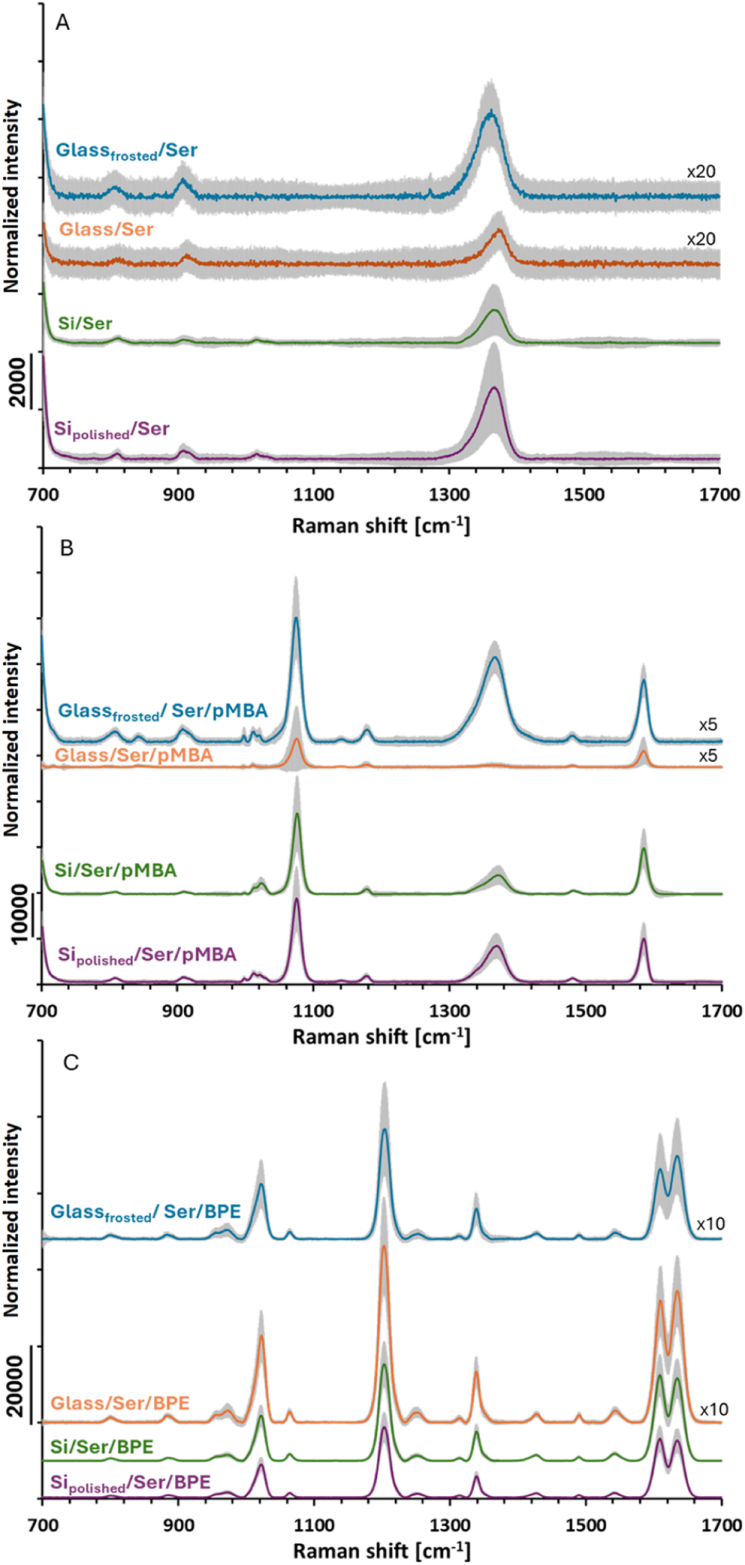
SERS spectra acquired for substrates made with AuNPs synthesized with l-serine. (A) Background spectra of clean substrates, (B) SERS spectra of pMBA, and (C) SERS spectra of BPE.

**Figure 8:**
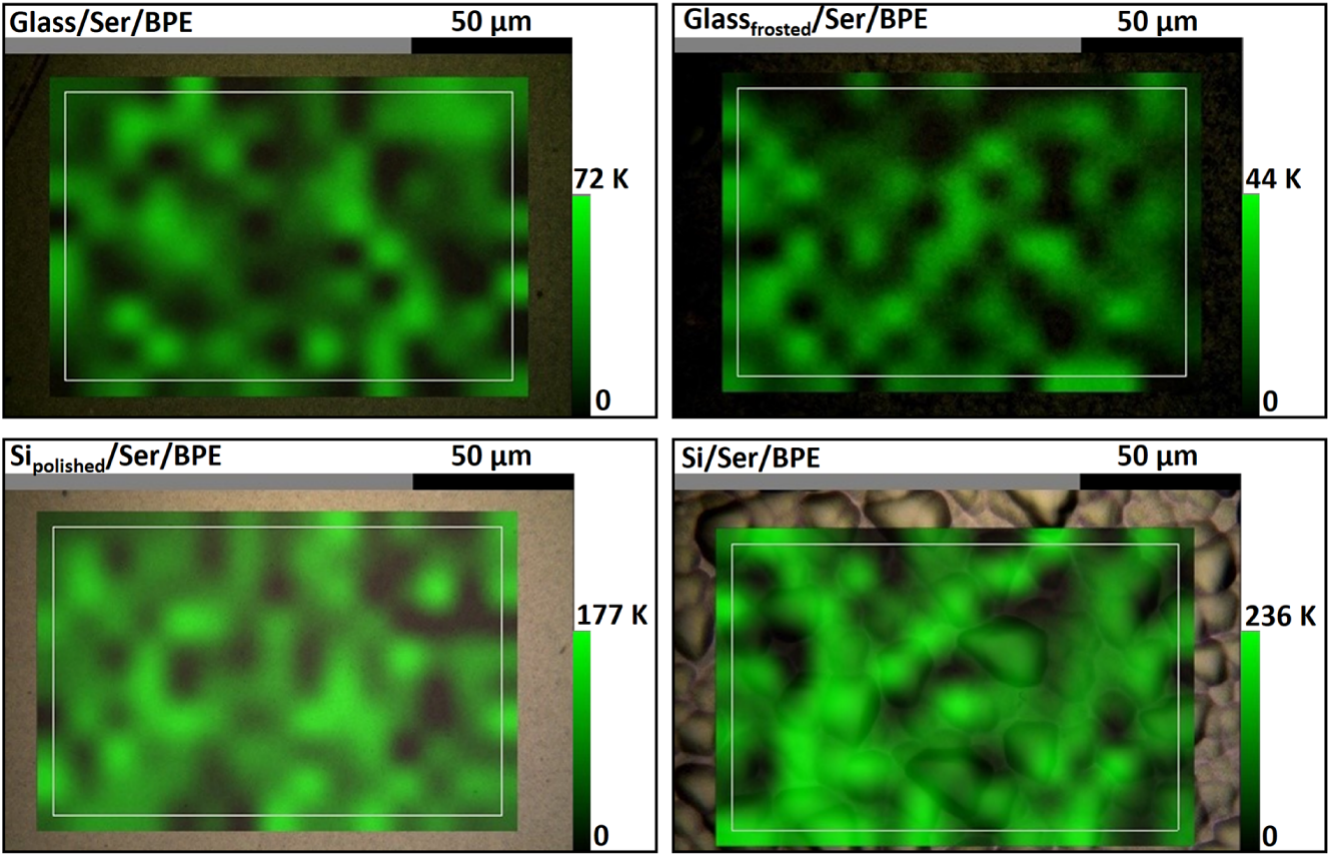
2D maps of BPE SERS signal intensity at 1200 cm^–1^ acquired for SERS substrates fabricated using Ser-synthesized AuNPs. SERS substrates were made using the following platforms: glass (upper left), frosted glass (upper right), polished silicon (bottom left), and silicon (bottom right).

The pMBA and BPE spectra acquired for SERS substrates made with Phe-synthesized AuNPs are shown in Figure S15 (Supporting Information). Additionally, the 2D map of BPE SERS intensity at 1200 cm^–1^ acquired to show the distribution of “hot spots” is shown in Figure S16 (Supporting Information). These SERS substrates do not show any additional peaks in their background spectra beyond those originating from glass or silicon. In the case of pMBA, SERS substrates fabricated on platforms with rough surfaces exhibited higher EF than those fabricated on platforms with smooth surfaces (Table 1). The EF was over 3× higher for rough than smooth glass platforms and 2× higher for rough than smooth Si platforms. The average EF of the SERS substrate fabricated on a rough Si platform is nearly 1.5× higher than that on a rough glass platform. The standard deviation of SERS substrates on both smooth platforms is smaller than on rough platforms because the enhancement comes evenly over the entire surface of the SERS substrate, and the maximum enhancement in the “hot spot” is only twice as high as the average. For SERS substrates on rough platforms, the best “hot spot” yields more than 3× higher EF than the average EF of the SERS substrate. In the case of BPE, the highest average and maximum EFs were obtained for the SERS substrate fabricated on a frosted glass platform, which were 2× higher than the EFs estimated for the SERS substrate fabricated on smooth glass. The SERS substrate fabricated on a rough Si platform had an average EF of over 2.5× lower than that of the SERS substrate fabricated on a smooth glass platform and 3× higher than that of the SERS substrate fabricated on a polished Si platform.

The pMBA and BPE spectra acquired for SERS substrates made with Val-synthesized AuNPs are shown in Figure S17. Additionally, the 2D map of BPE SERS intensity at 1200 cm^–1^ acquired to show the distribution of “hot spots” is shown in Figure S18. These SERS substrates do not show any additional peaks in their background spectra beyond those originating from glass or silicon. In the case of pMBA, SERS substrates fabricated on a rough Si platform exhibited the highest average EF among SERS substrates fabricated with Val-synthesized AuNPs. The standard deviation of the SERS substrate on a rough Si platform shows the even distribution of well-enhancing “hot spots” over the measured surface. SERS substrates on rough glass and Si platforms exhibited higher average EFs than those on smooth surfaces. The average EFs of SERS substrates made on glass platforms had similar values. However, in the case of the SERS substrate prepared on a smooth glass platform, the standard deviation is higher than the average EF, and the maximum EF is approximately 12 times higher than the average EF. It suggests that the average EF is highly increased by single well-enhancing “hot spots.” In the case of BPE, the highest average and maximum EFs were obtained for the SERS substrate fabricated on a smooth glass platform. As shown by the “hot spot” distribution map (Figure S18), several well-enhancing “hot spots” allowed us to obtain a higher average EF than uniformly distributed “hot spots” on the SERS substrates fabricated on a polished Si platform. Smooth glass platforms give 2× higher average and maximum EFs than frosted glass platforms. In the case of SERS substrates fabricated on Si platforms, the SERS substrate on a rough Si platform yields an average EF almost 2× lower than the EF for the SERS substrate on smooth glass but 2× higher than the SERS substrate on a polished Si platform.

The pMBA and BPE spectra and maps acquired for SERS substrates made with Lys-synthesized AuNPs are shown in Figures S19 and S20 (Supporting Information). The only substrate we were able to fabricate using Lys-synthesized AuNPs was on frosted glass. For this SERS substrate, estimated EFs for pMBA and BPE were 4.90 × 10^4^ and 1.72 × 10^5^, respectively (Table 1). For both Raman reporters, the average EFs obtained for this SERS substrate were higher than those of SERS substrates fabricated with Phe- and Val-synthesized AuNPs but lower than those of SERS substrates fabricated with Ser-synthesized AuNPs (Table 1 and Figure 6).

As mentioned in the section on substrate fabrication, substrates fabricated with Hyp-synthesized AuNPs were shown to be unsuitable for SERS due to the rich background spectrum and low-intensity SERS signals of reporters (Figure S21). Based on previous studies,^43^ AuNPs with a morphology similar to Hyp-synthesized AuNPs (Figure 2) should contribute to strong plasmon enhancement. Therefore, we expected good performance from SERS substrates made from them. However, in the background spectra and spectra measured after the deposition of reporters, the strong Raman bands that could not be assigned to pMBA and BPE were observed (Figure S21). The acquired background spectra differ from those observed for other fabricated substrates and show spectral features that closely resemble those reported for SERS of the Pro-Pro homodipeptide.^59^ These results indicate that during the synthesis of AuNPs, the Hyp, in addition to other reactions, undergoes peptidization.^60^ Based on the SERS studies of Pro-Pro peptides,^59^ we propose the following band assignments corresponding to Hyp-oligomers, likely Hyp-Hyp dipeptides. The band at approximately 730 cm^–1^ can correspond to δ­(COOH), γ­(CH_2_), Amide V, and/or *r*(CH_3_). The band at ca. 850 cm^–1^ can correspond to ν­(C–C), τ­(CH_2_), and/or *r*(NH_3_^+^). The band at ca. 940 cm^–1^ can be assigned to Hyp ring and/or ν­(C–COOH). The bands between ca. 980 and 1080 cm^–1^ can correspond to ν_as_(C–NH_3_^+^), τ­(NH_3_^+^), ω­(CH_2_), and/or ν_as_(C_α_–C–N). The band at ca. 1340 cm^–1^ can be assigned to ν_s_(COOH). Finally, the bands between ca. 1540–1640 cm^–1^ correspond to δ_s_(NH_3_^+^) and/or amide II, ν_as_(COOH), δ_as_(NH_3_^+^) and/or amide I. In the spectra measured after the deposition of pMBA and BPE, in addition to the bands described above, the bands that potentially correspond to these compounds were observed. In spectra acquired for BPE, the characteristic band at 1200 cm^–1^ is observed; however, the other characteristic bands at approximately 1020, 1600, and 1640 cm^–1^ overlap with the background bands. Similarly, in spectra acquired for pMBA, the characteristic band at 1075 cm^–1^ is observed; however, the other characteristic band at approximately 1585 cm^–1^ overlaps with the background bands. Due to the rich background spectrum of substrates fabricated with Hyp-synthesized AuNPs and low-intensity SERS signals of reporters, we were unable to estimate EFs for pMBA and BPE reliably.

In the section on SERS substrate fabrication, we discussed how the surface chemistry of AuNPs influences the fabrication of substrates. The surface chemistry of AuNPs can also influence the SERS performance of substrates fabricated using them. As discussed by the authors of recently reported studies,^73^ the chemical synthesis of metal nanomaterials with SERS-enhancing properties often involves the use of strongly adsorbing modifier molecules, which can passivate their surface. This may prevent the application of such nanomaterials in SERS analysis of weaker molecule–metal interactions. Therefore, chemical ligands on surface-accessible nanomaterials for use in SERS should be easily displaced by target molecules relevant to potential applications.^73^ Considering the similarities in the roles of amino acids and citrate in the synthesis of AuNPs as reducing and capping agents,^51^ as well as the molecular structures of the amino acids used in this study, we expected that molecules of the test analytes would easily displace these amino acids as it was observed for citrate in SERS substrates made with citrate-synthesized AuNPs.^31,40,73^ The specific case of Hyp-synthesized AuNPs was discussed in the previous paragraph. Taking into consideration the values of enhancement factors (EFs) obtained for SERS substrates fabricated with Ser-, Val-, Phe-, and Lys-synthesized AuNPs, we can infer that the molecules of pMBA and BPE easily displace them. Therefore, surface chemistry does not significantly influence the SERS performance of the substrates fabricated using AuNPs synthesized with these amino acids.

## Conclusions

In summary, we successfully fabricated SERS substrates by depositing amino acid-synthesized AuNPs on amino-functionalized smooth and rough glass and silicon platforms. The combination of AuNPs synthesized with five amino acids and four platforms yielded 17 SERS substrates. The evaluation of SERS properties of fabricated substrates by measuring the SERS spectra of pMBA and BPE molecules adsorbed on them showed that the highest EFs (2.10 × 10^5^ and 1.14 × 10^6^ for pMBA and BPE, respectively) were estimated for SERS substrates fabricated with AuNPs synthesized with l-serine (largest particle size). In most cases, SERS substrates made with a platform having a rough surface had a higher EF compared to corresponding SERS substrates made with a platform having a smooth surface. Notably, the surface chemistry of AuNPs was found to play a crucial role in their deposition on used platforms, but did not affect their SERS performance. The AuNPs synthesized with l-lysine, likely due to the relatively strong interaction of this amino acid with the surface of AuNPs, the ε-amino groups of Lys side chain extended out to the solvent, and their relatively large size, deposited only on frosted glass. SERS experiments revealed that AuNPs synthesized with Hyp are not capped by Hyp molecules but are instead bound and stabilized by the Hyp oligomers formed during the synthesis of AuNPs. The Hyp-oligomers contribute strongly to the background spectrum of substrates made from Hyp-synthesized AuNPs and limit adsorption and/or separate Raman reporters from “hot spots”, making such substrates unsuitable for SERS.

Due to the availability of components and the simplicity of fabrication, the SERS substrates described in this article can be easily fabricated by researchers or other potential users of the SERS technique, such as first responders. In our future work, we will focus on utilizing the best-performing substrates for detecting trace amounts of hazardous chemicals, such as explosive materials, and comparing them with commercially available or fabricated SERS substrates.

## Experimental Section

### Materials

The SERS substrates used in these studies were fabricated using glass slides, silicon wafers, and AuNPs. The microscope glass slides were purchased from JSHD Co. Ltd. (China). The frosted glass slides were obtained by polishing the surface of the glass slides with 3 μm alumina (Al_2_O_3_) powder (Schmitz-Metallographie GmbH, Germany). The particle size of the glass polishing powder was selected to achieve a fine surface roughness, enabling accurate surface measurements using microscopy. The obtained surface roughness was within the depth of the field of the microscope’s objective lens, allowing clear and precise imaging. This balance between adequate surface matting and controlled roughness made the used polishing powder ideal for our experiments. The polished n-type silicon wafer with orientation <100> was purchased from the Łukasiewicz – Institute of Microelectronics and Photonics (Poland), and both sides (smooth and rough) of the wafer were used as platforms for SERS substrates fabrication. Sulfuric acid (95%) and hydrogen peroxide (30%), used for the preparation of the piranha solution, were purchased from Chempur and Stanlab, respectively. The platform’s surface functionalization with amino groups was carried out using 0.3 M (3-aminopropyl)­trimethoxysilane (97%, Alfa Aesar) in toluene (99.5%, POCH Basic). After functionalization, the platforms were rinsed with technical-grade ethanol (Linegal Chemicals) and ultrapure deionized water (18.2 MΩ cm at 25 °C, Hydrolab, Poland). All materials used to synthesize AuNPs were identical to those used in our previous work.^51^ The SERS measurements were carried out using two test analytes, p-mercaptobenzoic acid (90%, Sigma-Aldrich) and 1,2-bis­(4-pyridyl)­ethylene (97%, Sigma-Aldrich).

### Fabrication of SERS Substrates

The SERS substrates used in these studies were fabricated using four platforms based on glass and silicon (Figures S1–S2 and Table S1). The glass platforms constituted the smooth surface of microscope glass slides assigned in our studies as Glass, and the frosted surface of microscope glass slides assigned in our studies as Glass_frosted_. The silicon platforms constituted the silicon wafer’s smooth (Si_polished_) and rough (Si) surfaces. Platforms were cleaned in piranha solution (H_2_SO_4_:H_2_O_2_ 3:1), washed with water and acetone, and then functionalized with 0.3 M (3-aminopropyl)­trimethoxysilane in toluene at 100 °C for 1 h. After rinsing in ethanol and water, the slides were placed at the bottom of a Petri dish containing a suspension of AuNPs for at least 12 h, with continuous stirring. Such fabricated SERS substrates were thoroughly rinsed with water to remove contaminants from the AuNPs solution and left to dry under a cover at ambient temperature. Gold nanoparticles used in these studies were synthesized using l-valine (Val), dl-phenylalanine (Phe), l-(4)-hydroxyproline (Hyp), l-serine (Ser), and l-lysine (Lys) using the same experimental procedure described in our previous work.^51^ Briefly, 3 mL of a 5 mM HAuCl_4_ aqueous solution was added to a 250 mL flask containing 54 mL of water, and the solution was brought to a boil while stirring magnetically at 400 rpm. Then, 3 mL of a 20 mM α-amino acid salt aqueous solution was added at once. The reaction was carried out until the solution acquired a color that remained unchanged for an additional 5 min. Seventeen different SERS substrates were obtained by depositing five types of AuNPs with different plasmonic properties (Table S4) on four platforms.

### Characterization of AuNPs and SERS Substrates

The imaging and elemental analysis of AuNPs were performed using a Talos F200X FEI transmission electron microscope (Thermo Fisher Scientific) equipped with the Super-X EDS system, featuring four windowless single silicon drift detectors (Bruker) at an accelerating voltage of 200 kV. The samples were deposited on 300-mesh carbon-coated copper grids (Pacific Grid).

The chemical composition of the platforms’ surface (after cleaning and functionalization) and final SERS substrates was investigated using an X-ray photoelectron spectroscopy (XPS) spectrometer (PREVAC, Poland). The measurements were performed using an X-ray source with an Al anode emitting X-ray radiation with a photon energy of 1486.6 eV. The registered XPS spectra were analyzed using CasaXPS software.

The platforms’ morphology was investigated using an atomic force microscope (AFM) (NT-MDT Spectrum Instruments, Russia). AFM enabled imaging of the platforms’ surface topography and determination of their surface roughness. The AFM measurements were conducted in a semicontact mode under ambient conditions using a silicon AFM probe (HQ:NSC15/Al BS, MikroMasch, Innovative Solutions, Bulgaria). The measurements were conducted in two distinct areas on the surface of each platform. The average and root-mean-square roughness were then determined for the examined areas. The morphology of fabricated SERS substrates was analyzed using a scanning electron microscope (SEM) (Quanta 3D FEG, FEI, Eindhoven, The Netherlands). The SEM images were taken from a larger area of the substrates to show the distribution of nanoparticles.

The wettability of the SERS substrates’ surfaces was determined by measuring the water contact angles (WCA). The measurements were performed using a Mobile Surface Analyzer (Krüss, Hamburg, Germany) with the software Advance at ambient conditions and room temperature. The volume of the water droplets for the WCA measurements was 2 μL, and they were released over 1 s. The final WCA values are the average for five surface measuring points.

The optical properties of SERS substrates were investigated using a Lambda 650 UV–vis spectrophotometer (PerkinElmer, Waltham, MA, USA). A 150 mm integrating sphere was used to examine the glass-based SERS substrates in transmittance mode using a center-mount sample holder, while the silica-based substrates were measured only in reflectance mode. The samples were measured within the 250–850 nm spectral range with a 1 nm increment. However, in the article, spectra are shown in the 450–850 nm spectral range, because below 450 nm only absorptance bands of the used platforms were observed. The instrument was calibrated using a Spectralon Diffuse Reflectance Standard.

### Raman and SERS Measurements

The Raman and SERS measurements were performed using a Renishaw inVia Reflex Raman microscope equipped with an EMCCD detector (Andor Technology Ltd., Oxford Instruments, Belfast, UK). The Raman signal was acquired using a continuous-wave (CW) laser with a wavelength of 785 nm and a nominal laser power of 60 mW incident on the sample. The laser beam was directed to the sample through an objective lens with a magnification of 50× (N.A. = 0.75). The estimated laser spot diameter at these measurement parameters was ca. 2 μm. The measurement parameters used for Raman measurements of SERS substrates and SERS measurements of p-mercaptobenzoic acid (pMBA) and 1,2-bis­(4-pyridyl)­ethylene (BPE) are presented in Tables S5–S7. The measurement parameters were optimized for each substrate to achieve the highest intensities. SERS maps (140 × 90 μm) were acquired using a 50× objective with NA = 0.75, a step of 10 μm (15 × 10 points) and 1 s accumulation per point. The instrument was calibrated using an internal silicon wafer, and the spectrum was centered at 520.5 cm^–1^. The spectra of pMBA were obtained by averaging the SERS spectra from at least 100 measurement points from each of the three samples, which were fabricated in a similar manner. The averaged BPE spectra were obtained from at least 100 points from one SERS substrate. The SERS spectra were processed in WiRE 5.5 software. Then, the spectra were averaged using CasaXPS software.

All SERS substrates were used only once. The SERS measurements were performed on SERS substrates, which were first embedded in a 0.001 M solution of pMBA in ethanol or a 0.001 M solution of BPE in water. The substrates were then thoroughly washed with ethanol and dried in air. The detailed procedure for depositing the analyte on the SERS substrate has been described elsewhere.^74^ The enhancement factors (EFs) of all SERS substrates were estimated using eq 1, following the approach described in the Supporting Information.^67^

## Supplementary Material



## Data Availability

“Replication data for monolayers of amino acid-synthesized gold nanoparticles as SERS substrates for trace chemical sensing” is available at 10.18150/AWOS5Q.

## Notes

Photographs, microscopic images, AFM images, a table of AFM results, a table of SERS measurement parameters, EDX spectra, XPS spectra, a table of XPS results, contact angle measurement results, SEM images, SERS spectra, and 2D SERS maps (PDF)

## Abbreviations

AuNPs: gold nanoparticles
EF: enhancement factor
NPs: nanoparticles
SEM: scanning electron microscopy
SERS: surface-enhanced Raman spectroscopy.
