# Supplementary material for: Monolayers of Amino Acid-Synthesized Gold Nanoparticles as SERS Substrates for Trace Chemical Sensing
Source: Langmuir. 2025 Jul 22;41(30):19706–19. doi: 10.1021/acs.langmuir.5c01335 (PMC12333418; doi:10.1021/acs.langmuir.5c01335)
Supplement: Supplementary file 1 [file la5c01335_si_001.pdf]

Supporting Information

Monolayers of amino acid-synthesized gold nanoparticles as SERS substrates for trace chemical sensing

Aleksandra M. Figat,[a] *Malwina Liszewska,*[a] *Bogusław Budner,*[a] *Bartosz Bartosewicz,*[a] *Małgorzata Norek,*[b] *Bartłomiej J. Jankiewicz**[,a]

[a]Institute of Optoelectronics, Military University of Technology, gen. Sylwestra Kaliskiego 2, 00-908 Warsaw, Poland

[b]Faculty of Advanced Technologies and Chemistry, Military University of Technology, gen. Sylwestra Kaliskiego 2, 00-908 Warsaw, Poland

***Corresponding author contact information:**

Bartłomiej J. Jankiewicz

Institute of Optoelectronics, Military University of Technology, gen. Sylwestra Kaliskiego 2, 00-908 Warsaw, Poland

E-mail: bartlomiej.jankiewicz@wat.edu.pl

Phone: +48 261 837 639; Fax: +48 22 666 8950

Table of Contents

Title	Page
Photographs, microscopic photographs, and AFM images of platforms used for SERS substrates fabrication.	S3-S4
The average and root mean square roughness of the investigated platforms.	S4
EDX spectra of gold nanoparticles synthesized with amino acids.	S5-S7
XPS spectra of glass and silicon platforms after the cleaning procedure and functionalization.	S8-S9
XPS spectra of SERS substrate based on polished silicon platform and L-serine-synthesized AuNPs.	S9
The results of XPS measurements of SERS substrate based on amino-acid synthesized AuNPs deposited on different glass and silicon platforms.	S10
The measured contact angle of the investigated platforms and SERS substrates.	S11
SEM images of SERS substrates fabricated using gold nanoparticles.	S12-S15
SERS spectra and SERS maps of BPE.	S16-S21
The absorbance at the LSPR maximum of AuNPs utilized for the study.	S22
The parameters of Raman measurements of SERS substrates and SERS measurements of pMBA/ BPE on various SERS substrates.	S23
Procedure for evaluation of the total enhancement factor (EF)	S24

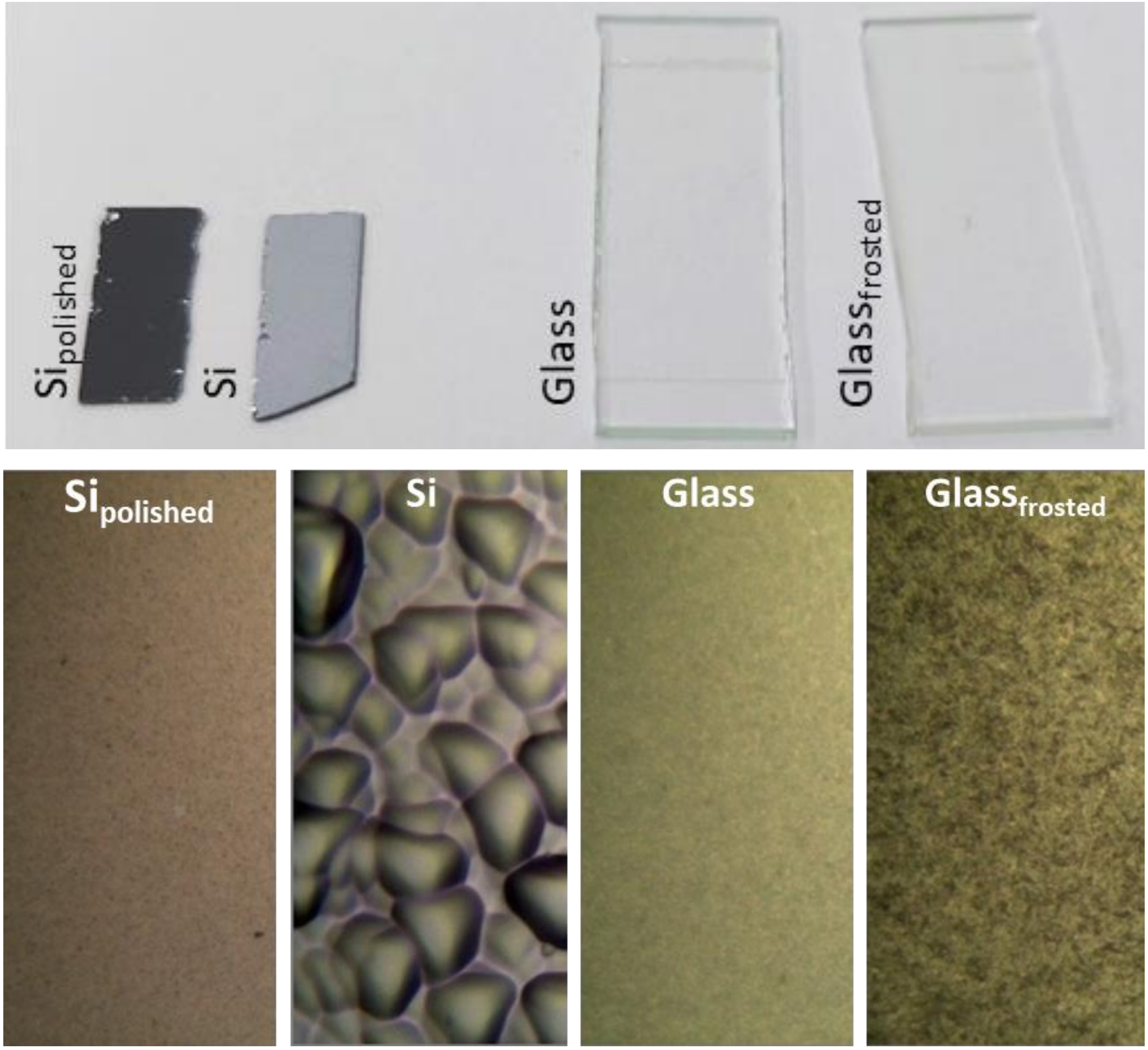

Figure S1. Photographs and microscopic photographs (objective 50×) of platforms used for SERS substrates fabrication.

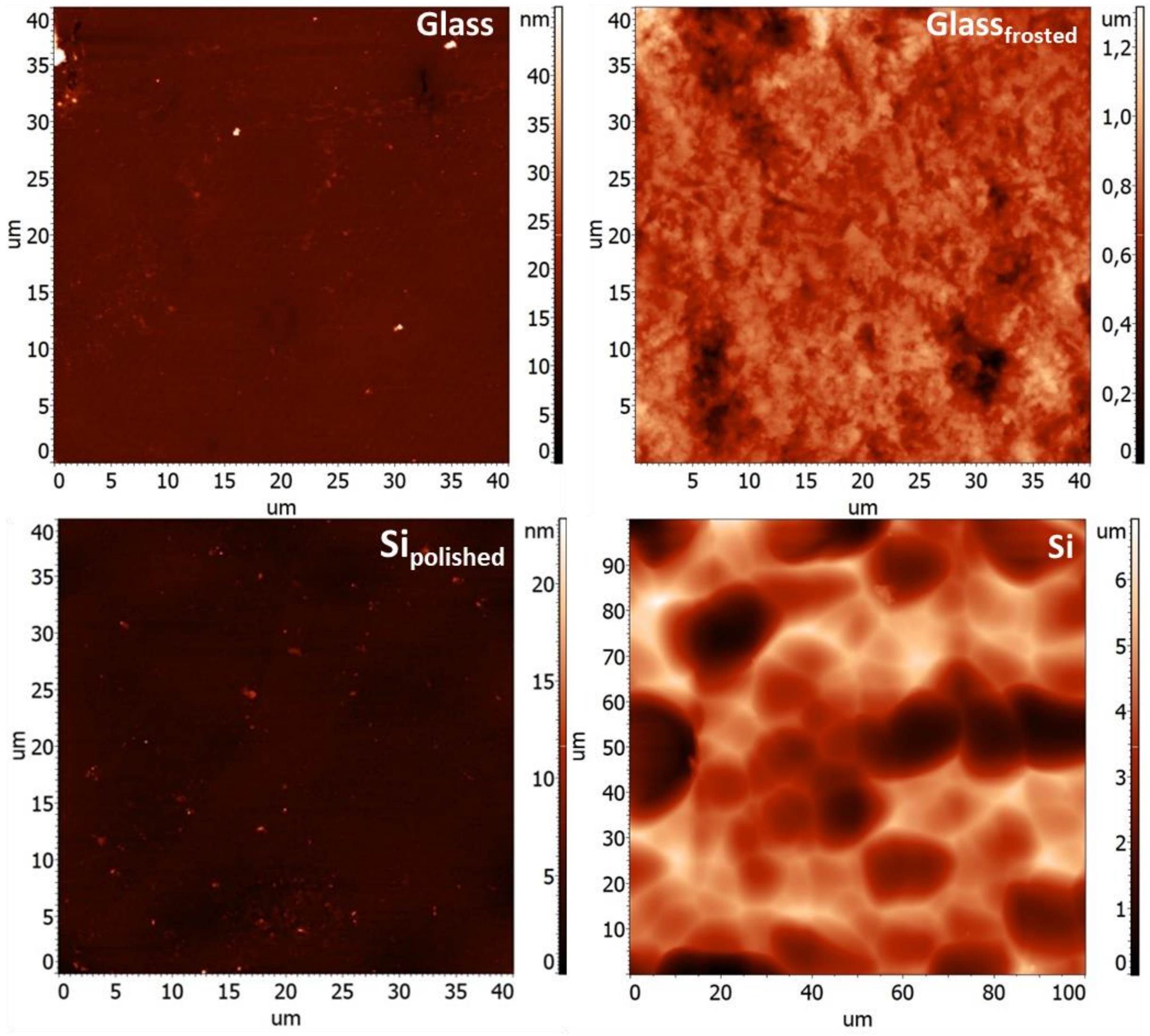

Figure S2. AFM images of surfaces of platforms used for SERS substrates fabrication.

Table S1. The average and root mean square roughness of the investigated platforms.

Platform	Average roughness [nm]	Root mean square roughness [nm]
Glass	0.5	1.1
Glass$_{frosted}$	98.4	131.5
Si$_{polished}$	0.6	1.9
Si	0.9	1.2

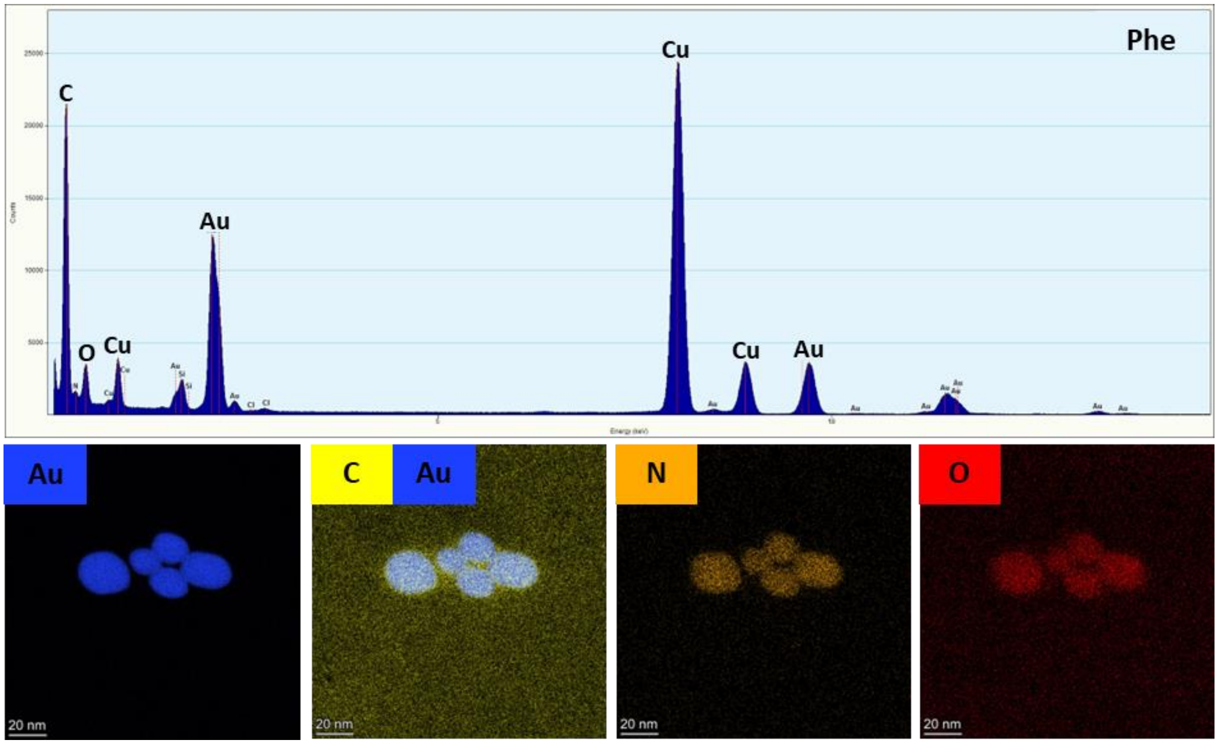

Figure S3. EDX spectra of gold nanoparticles synthesized with DL-phenylalanine.

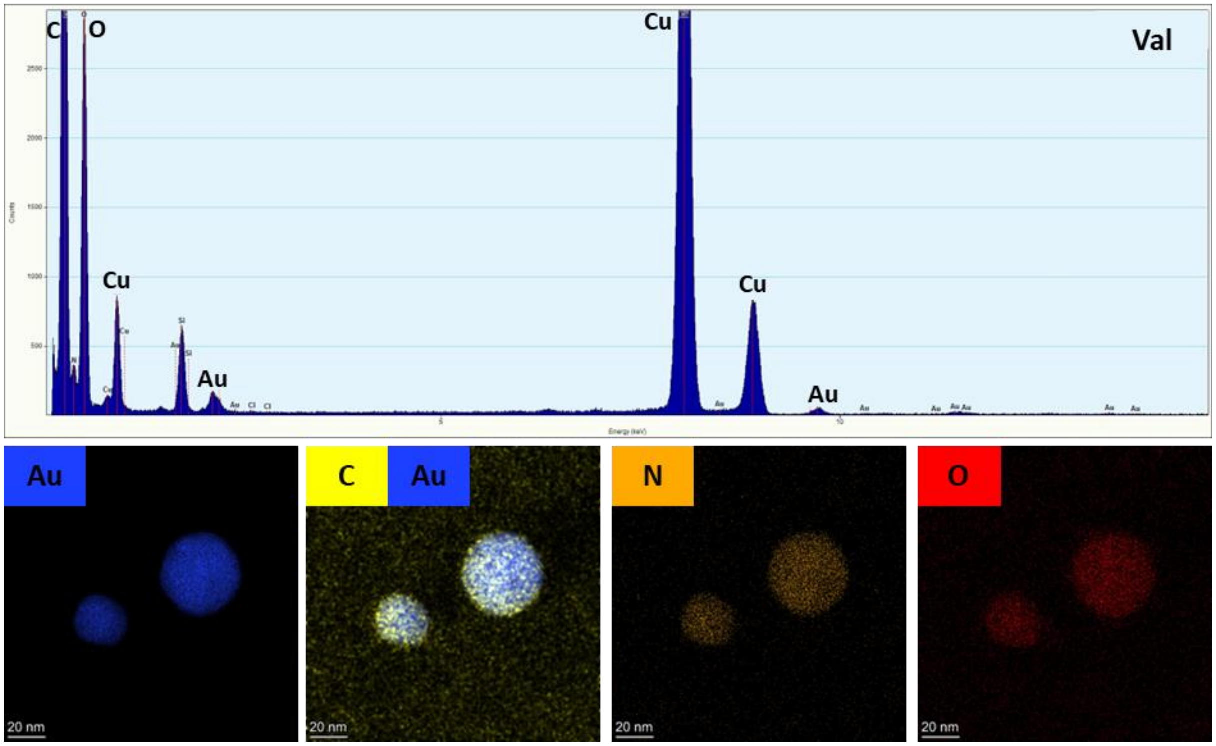

Figure S4. EDX spectra of gold nanoparticles synthesized with L-valine.

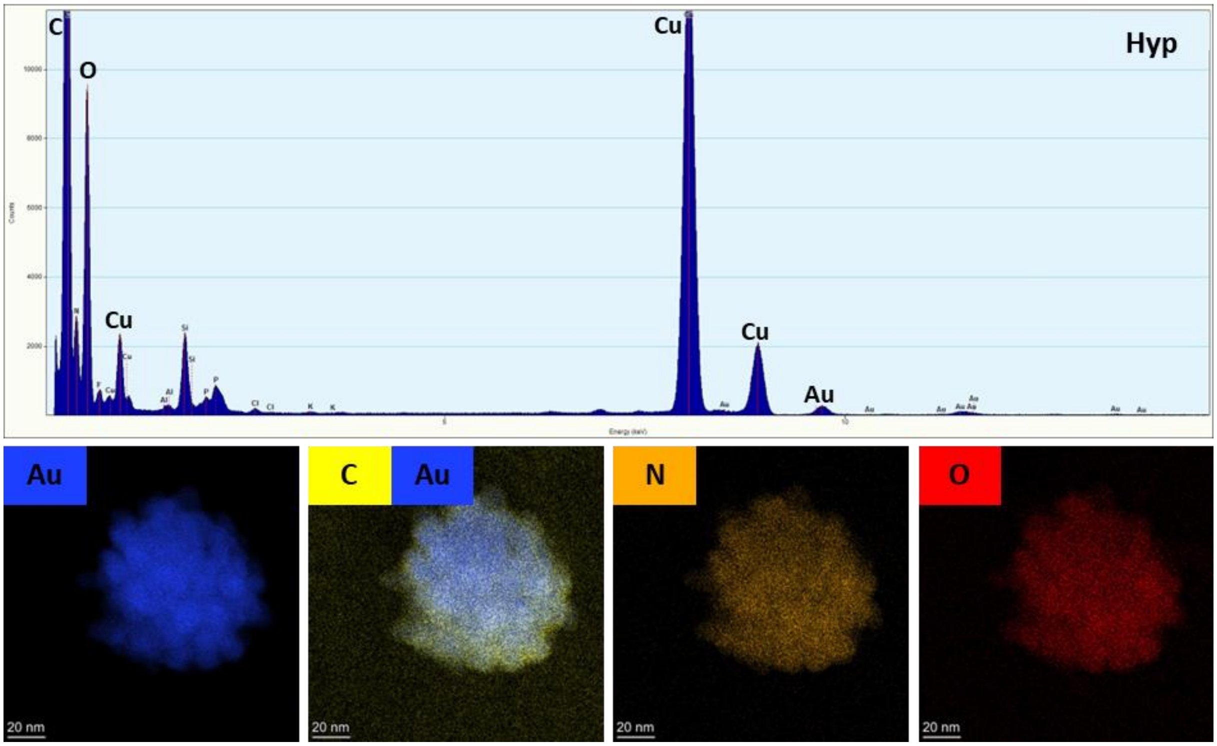

Figure S5. EDX spectra of gold nanoparticles synthesized with L-(4)-hydroxyproline.

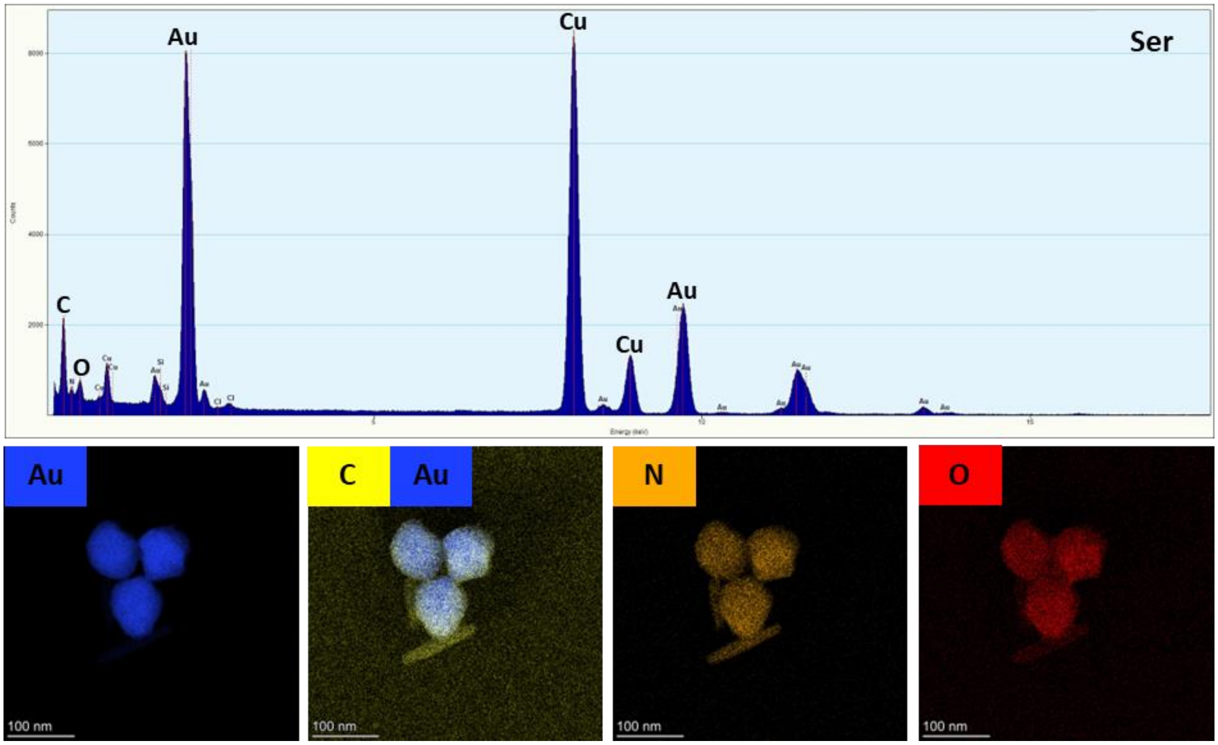

Figure S6. EDX spectra of gold nanoparticles synthesized with L-serine.

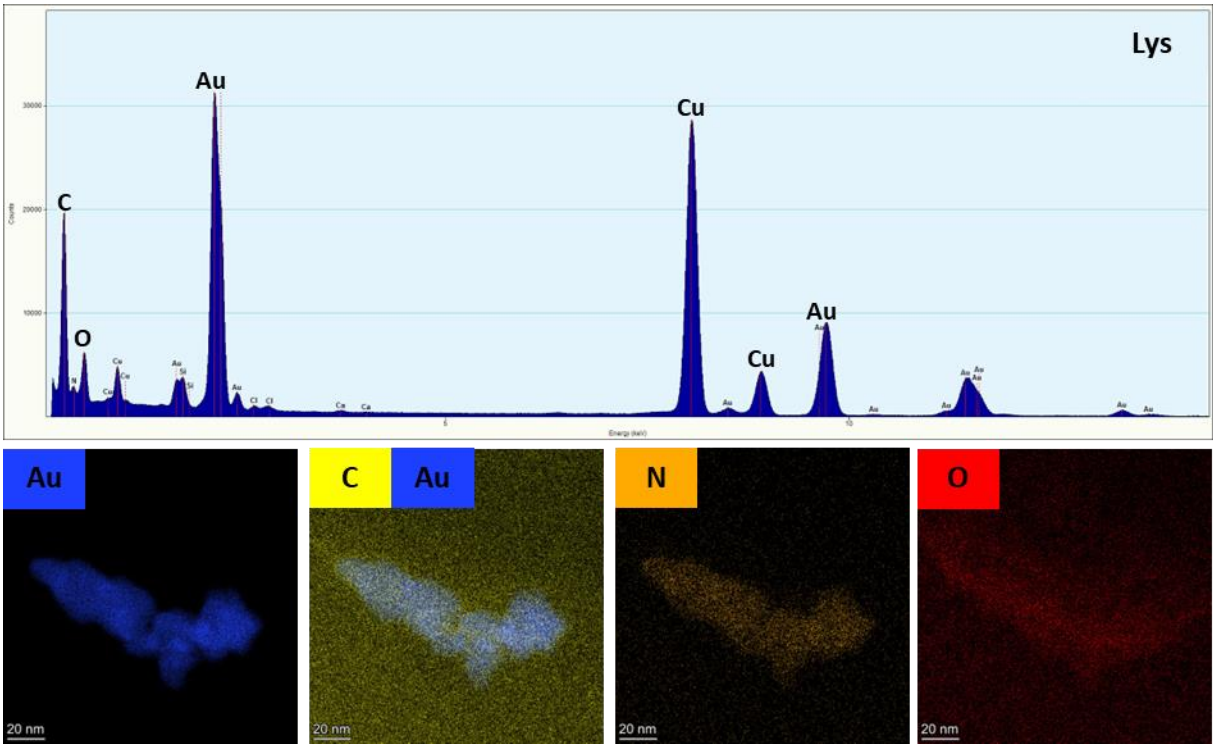

Figure S7. EDX spectra of gold nanoparticles synthesized with L-lysine.

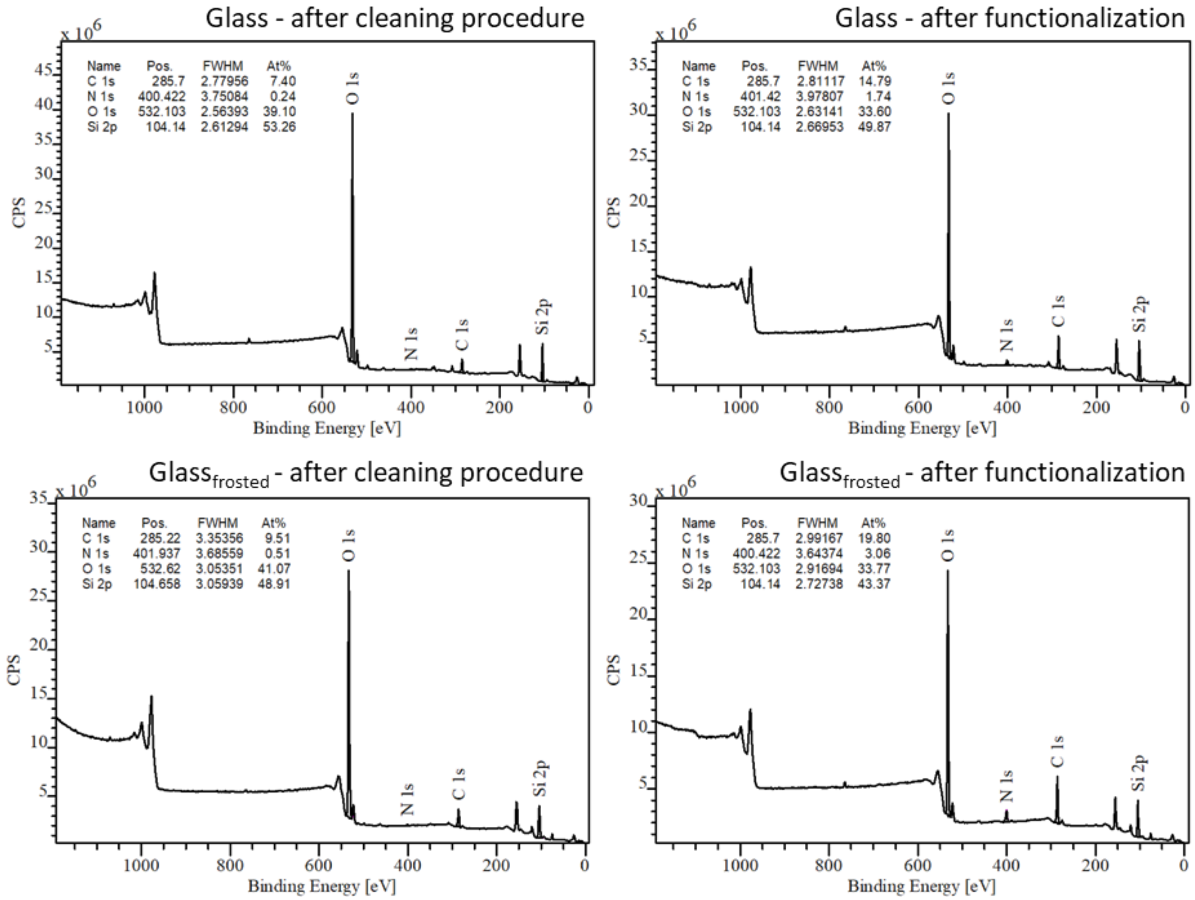

Figure S8. XPS spectra of glass platform after cleaning procedure and functionalization.

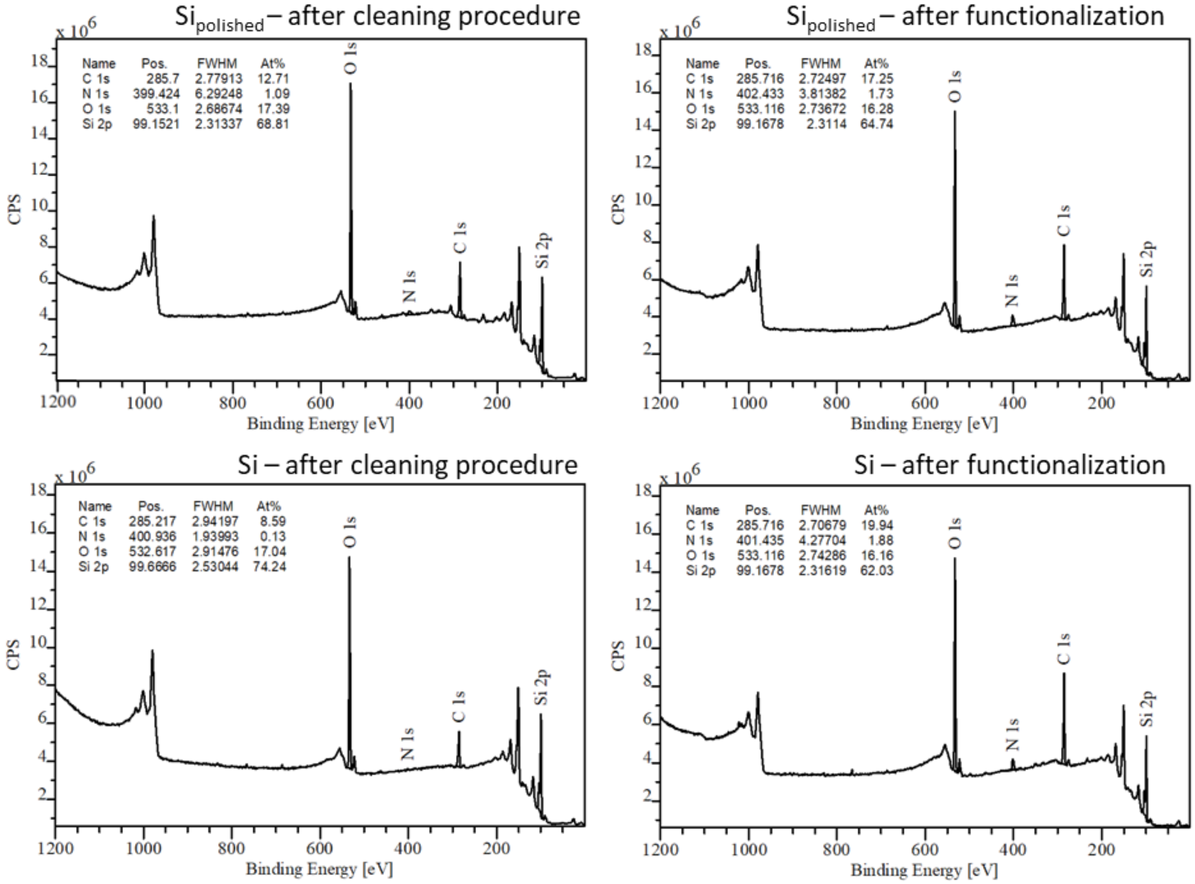

Figure S9. XPS spectra of silicon platform after cleaning procedure and functionalization.

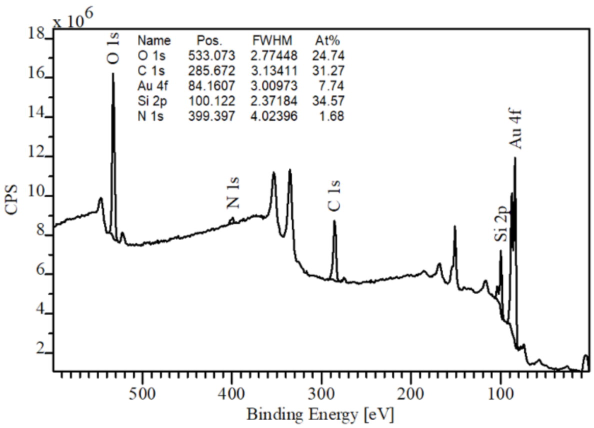

Figure S10. XPS spectra of SERS substrate based on polished silicon platform and L-serine-synthesized AuNPs.

Table S2. The results of XPS measurements of SERS substrate based on amino-acid synthesized AuNPs deposited on different glass and silicon platforms.

SERS substrate<br>Platform/Reductant in AuNPs synthesis	O [at%]	N [at%]	C [at%]	Si [at%]	Au [at%]
**$Si_{polished}$/Phe**	23.6	1.3	43.1	26.1	6.0
**$Si_{polished}$/Val**	24.8	1.9	37.9	27.7	7.7
**$Si_{polished}$/Hyp**	25.7	3.2	34.3	33.2	3.6
**$Si_{polished}$/Ser**	24.7	1.7	31.3	34.6	7.7
**Si/Phe**	23.3	1.5	45.1	24.0	6.2
**Si/Val**	25.4	1.7	36.2	28.4	8.2
**Si/Hyp**	24.2	2.9	36.5	33.2	2.9
**Si/Ser**	25.1	2.7	33.0	31.1	8.1
**Glass/Phe**	31.7	0.8	44.0	18.4	5.2
**Glass/Val**	39.7	1.1	31.2	21.4	6.6
**Glass/Hyp**	35.1	2.2	41.1	20.9	0.8
**Glass/Ser**	36.9	2.9	27.1	23.9	9.2
**$Glass_{frosted}$/Phe**	32.7	3.2	40.8	16.5	6.8
**$Glass_{frosted}$/Val**	37.9	1.6	31.7	21.2	7.6
**$Glass_{frosted}$/Hyp**	37.2	4.4	38.6	14.9	4.9
**$Glass_{frosted}$/Ser**	34.5	2.9	35.5	18.5	8.7
**$Glass_{frosted}$ /Lys**	41.3	2.0	30.6	19.7	6.4

Table S3. The measured contact angle of the investigated platforms and SERS substrates.

	Platform	Glass CA [°]	$Glass_{frosted}$ CA [°]	$Si_{polished}$ CA [°]	Si CA [°]
	After cleaning	39±2	22±1	29±2	31±4
	After functionalization	53±1	49±1	51±5	48±1
Deposited NPs synthesized with:	Phe	49±1	71±5	62±4	65±3
	Val	46±1	50±2	65±2	57±4
	Hyp	53±2	27±2	40±4	30±2
	Ser	44±2	66±1	55±4	59±2
	Lys	No deposition	37±3	No deposition	

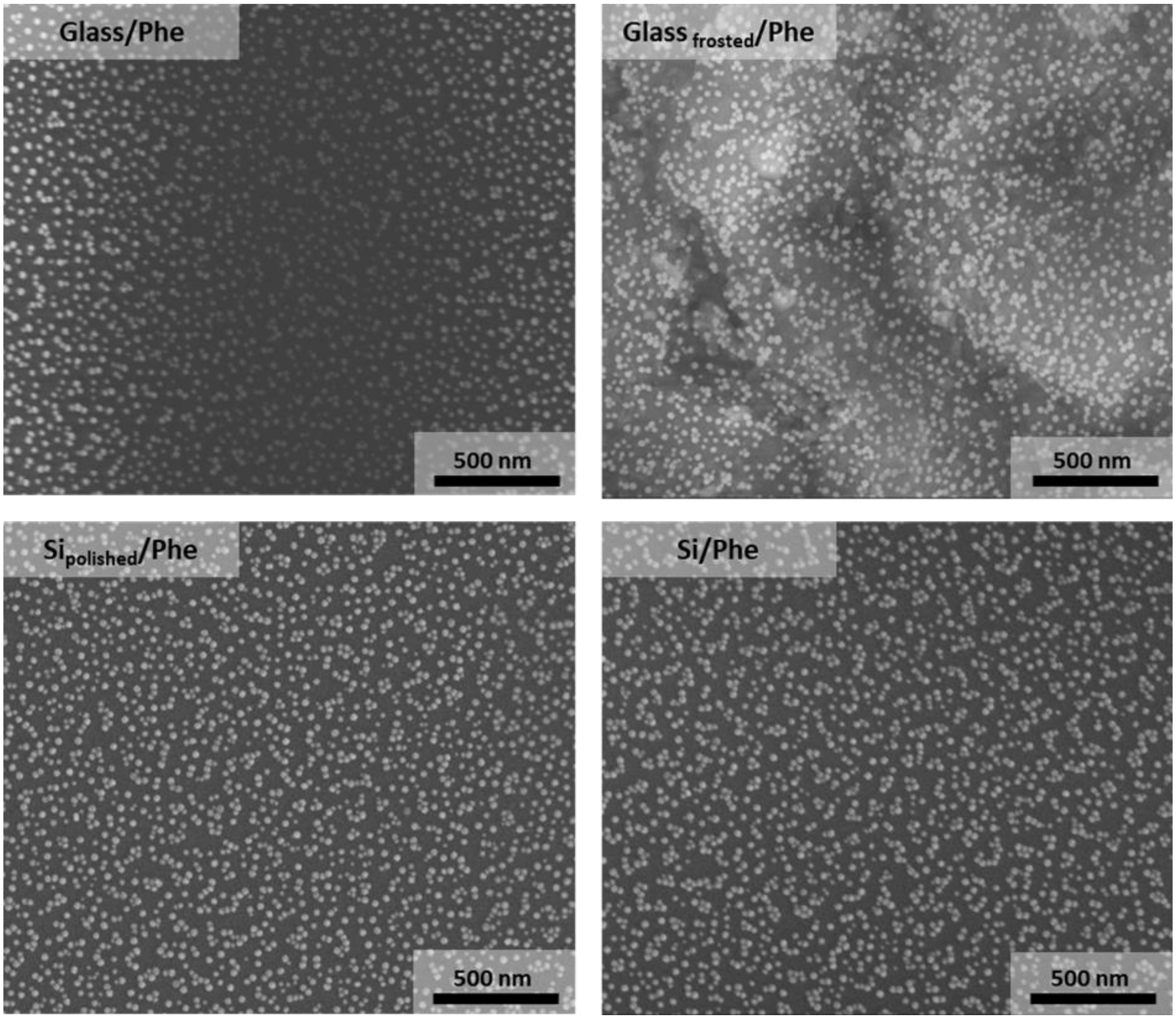

Figure S11. SEM images of SERS substrates fabricated using AuNPs synthesized with DL-phenylalanine. SERS substrates were made using the following platforms: glass (upper left), frosted glass (upper right), polished silicon (bottom left), and silicon (bottom right).

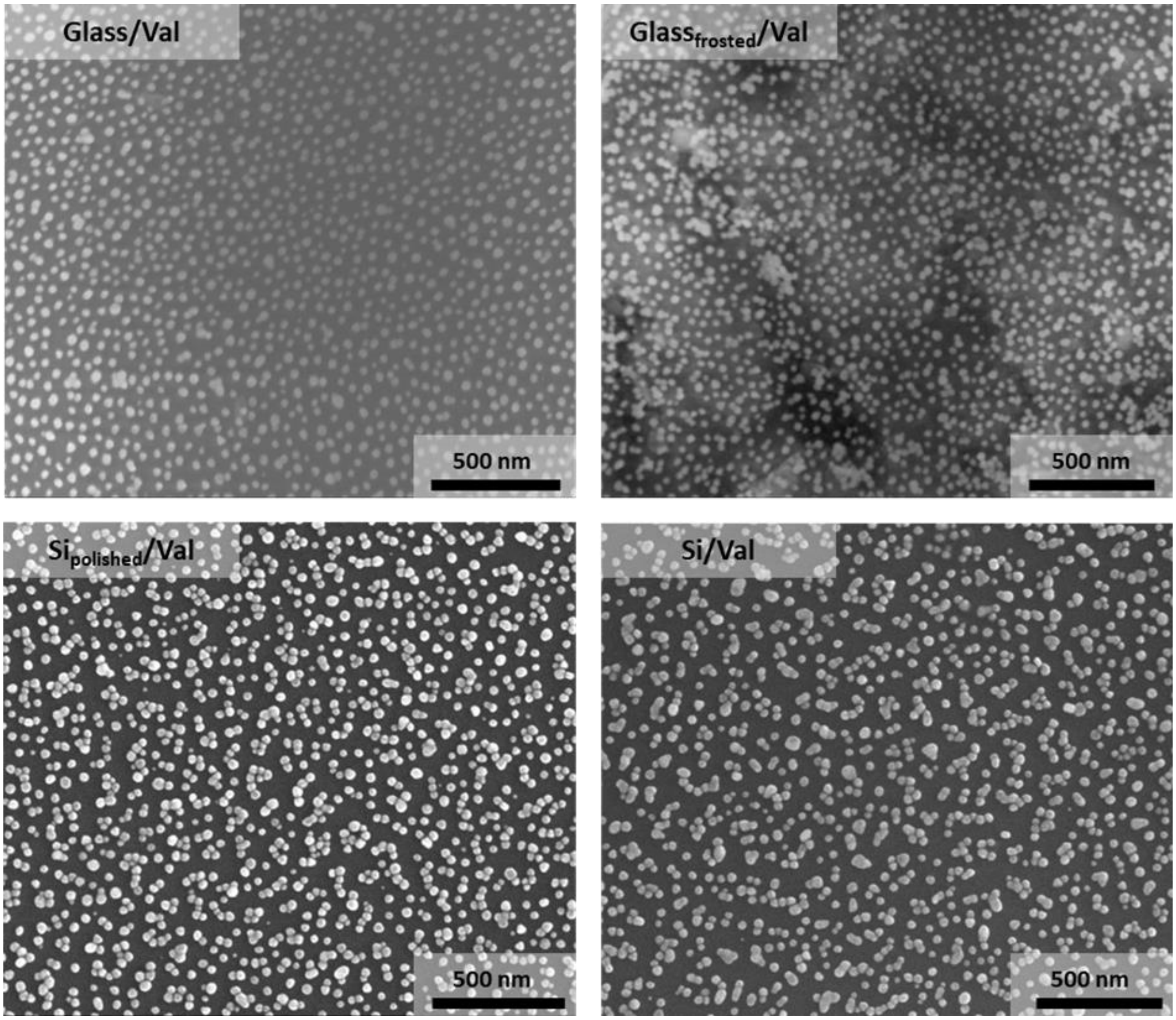

Figure S12. SEM images of SERS substrates fabricated using AuNPs synthesized with L-valine. SERS substrates were made using the following platforms: glass (upper left), frosted glass (upper right), polished silicon (bottom left), and silicon (bottom right).

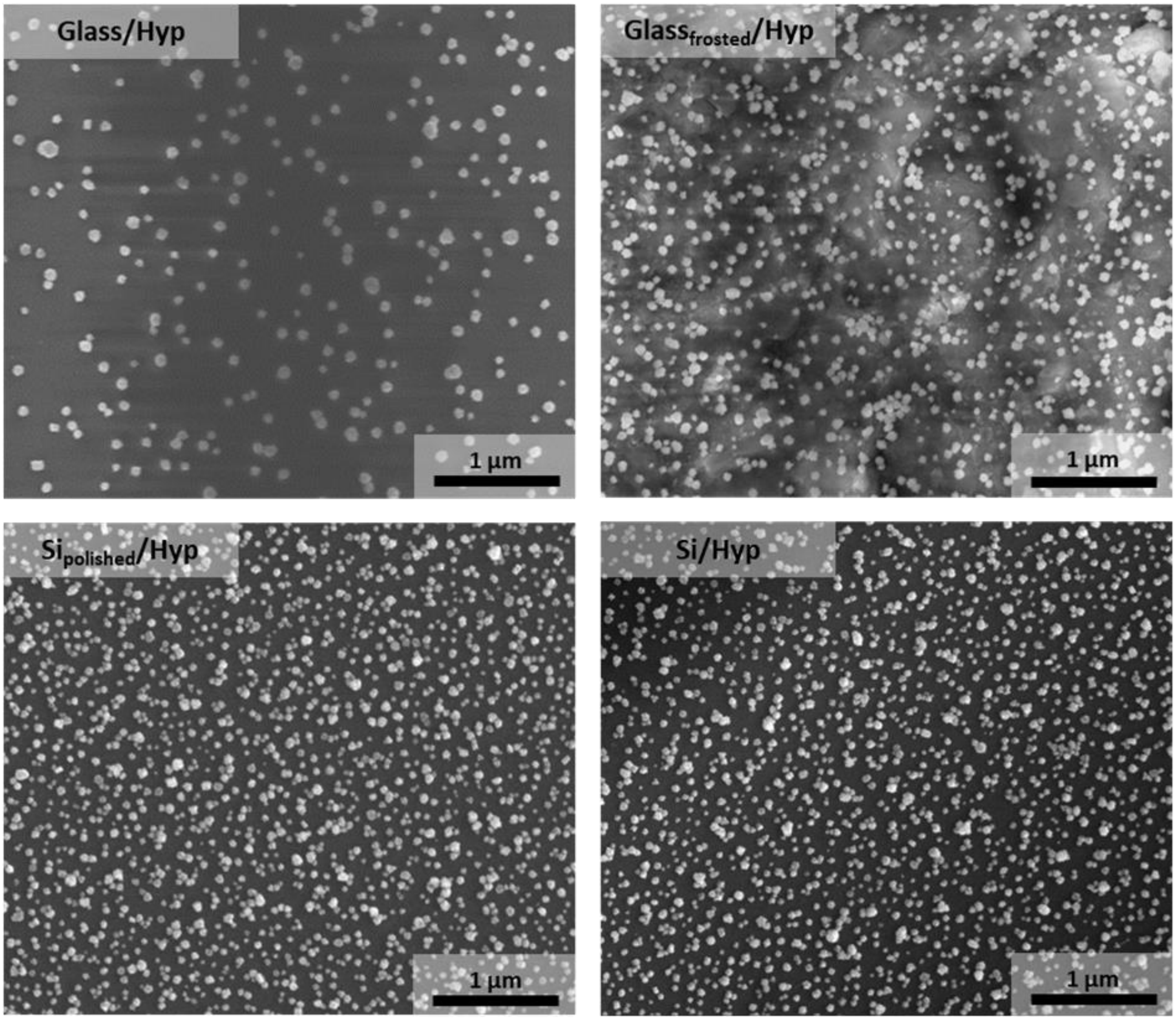

Figure S13. SEM images of SERS substrates fabricated using AuNPs synthesized with L-(4)-hydroxyproline. SERS substrates were made using the following platforms: glass (upper left), frosted glass (upper right), polished silicon (bottom left), and silicon (bottom right).

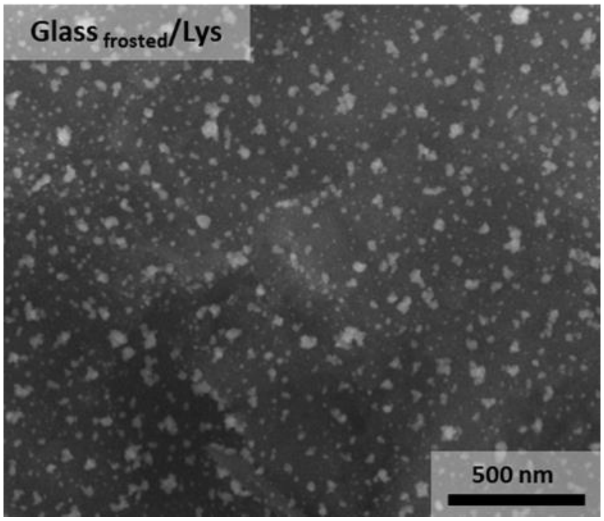

Figure S14. SEM image of SERS substrate fabricated using AuNPs synthesized with L-lysine. SERS substrate was made using frosted glass as a platform. The gold nanoparticles synthesized with L-lysine do not deposit on platforms other than frosted glass.

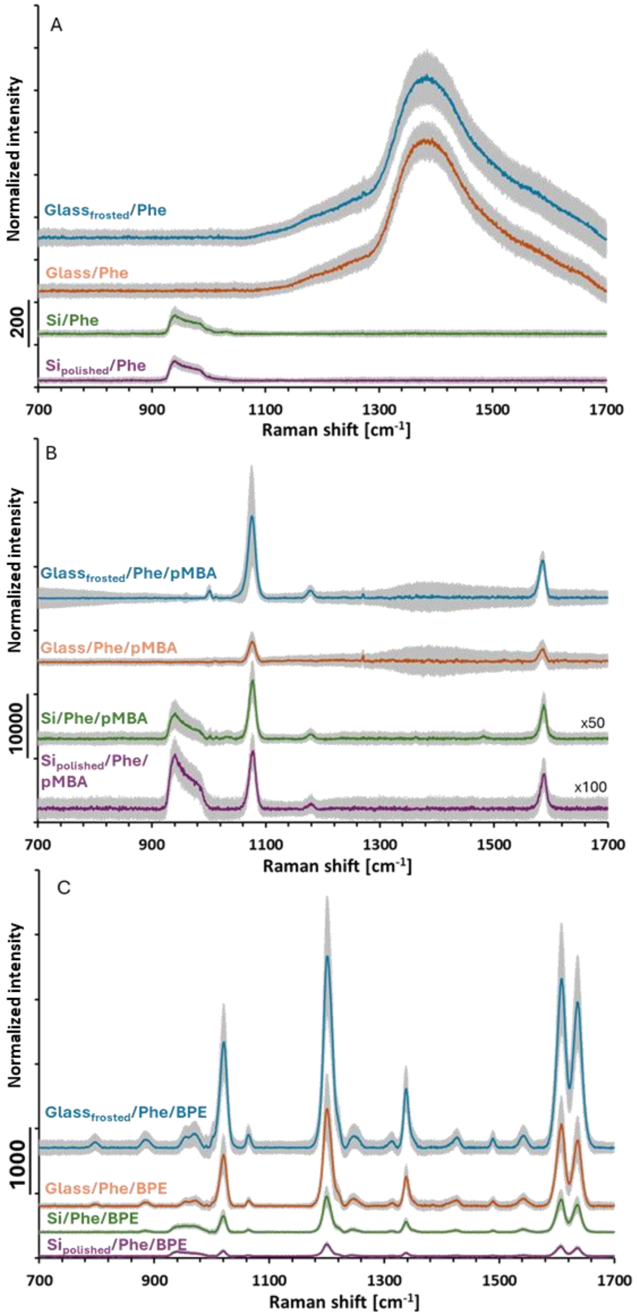

Figure S15. SERS spectra acquired for substrates made with AuNPs synthesized with DL-phenylalanine. A) Spectra of clean SERS substrates, B) SERS spectra of pMBA, C) SERS spectra of BPE. The gray shadow represents the standard deviation and was calculated using CasaXPS software based on over 300 measurements performed on three identically manufactured SERS substrates.

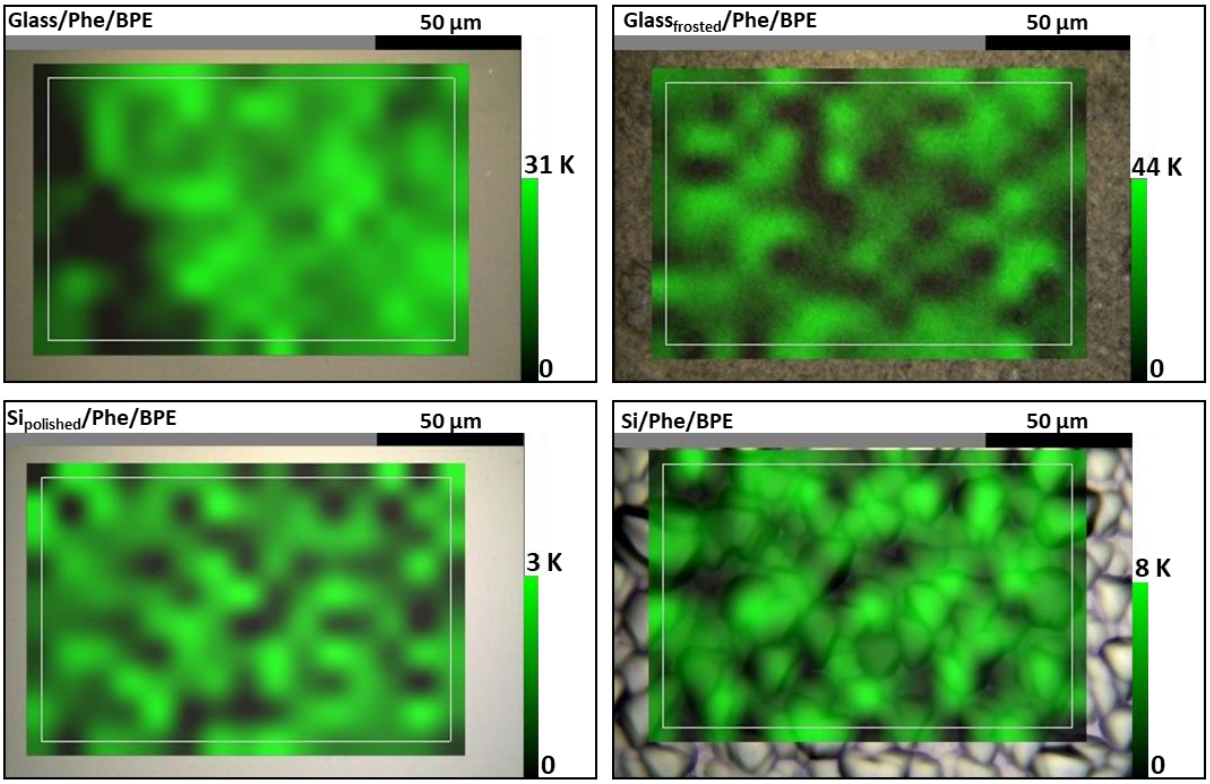

Figure S16. 2D maps of BPE SERS signal intensity at 1200 cm^{-1} acquired for SERS substrates fabricated using AuNPs synthesized with DL-phenylalanine. SERS substrates were made using the following platforms: glass (upper left), frosted glass (upper right), polished silicon (bottom left), and silicon (bottom right).

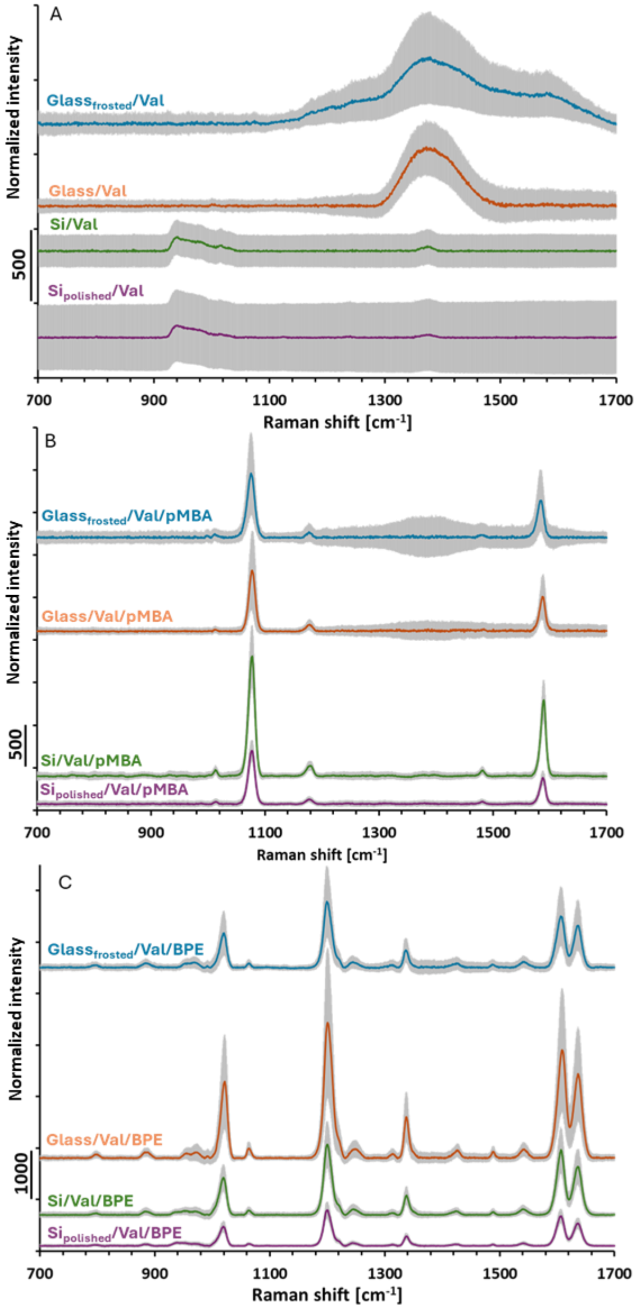

Figure S17. SERS spectra acquired for substrates made with AuNPs synthesized with L-valine. A) Spectra of clean SERS substrates, B) SERS spectra of pMBA, C) SERS spectra of BPE. The gray shadow represents the standard deviation and was calculated using CasaXPS software based on over 300 measurements performed on three identically manufactured SERS substrates.

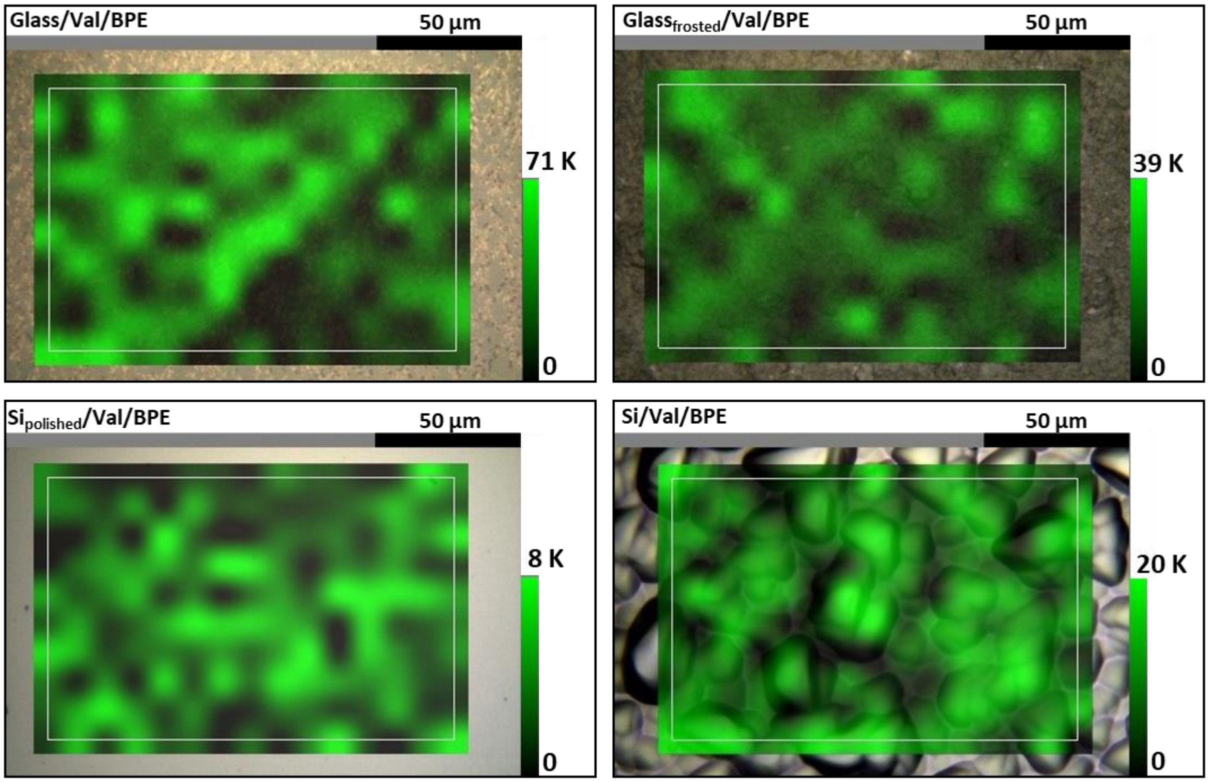

Figure S18. 2D maps of BPE SERS signal intensity at 1200 cm^{-1} acquired for SERS substrates fabricated using AuNPs synthesized with L-valine. SERS substrates were made using the following platforms: glass (upper left), frosted glass (upper right), polished silicon (bottom left), and silicon (bottom right).

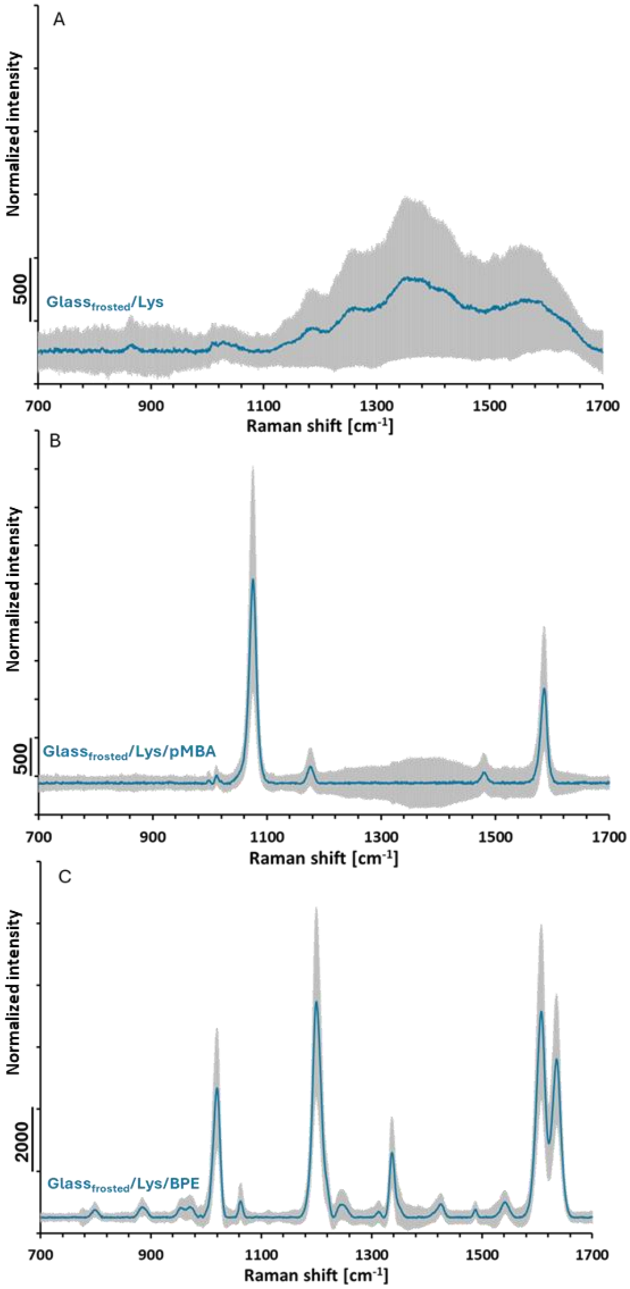

Figure S19. SERS spectra acquired for substrates made with AuNPs synthesized with L-lysine. A) Spectra of clean SERS substrates, B) SERS spectra of pMBA, C) SERS spectra of BPE. The gray shadow represents the standard deviation and was calculated using CasaXPS software based on over 300 measurements performed on three identically manufactured SERS substrates.

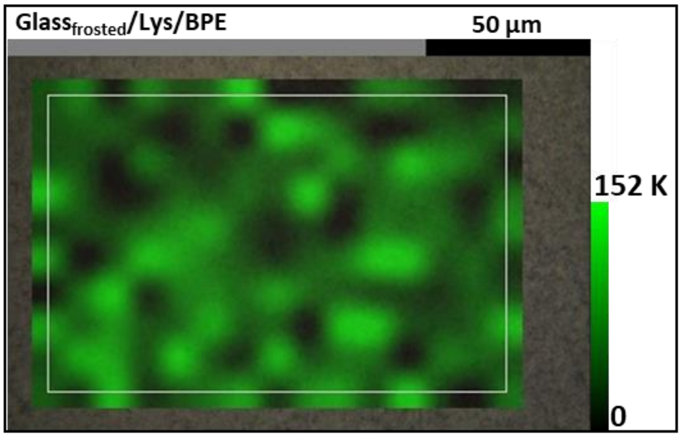

Figure S20. A 2D map of BPE SERS signal intensity at 1200 cm^{-1} acquired for SERS substrates fabricated using AuNPs synthesized with L-lysine. The SERS substrate was made using frosted glass as a platform. Gold nanoparticles synthesized with L-lysine do not deposit on platforms other than frosted glass.

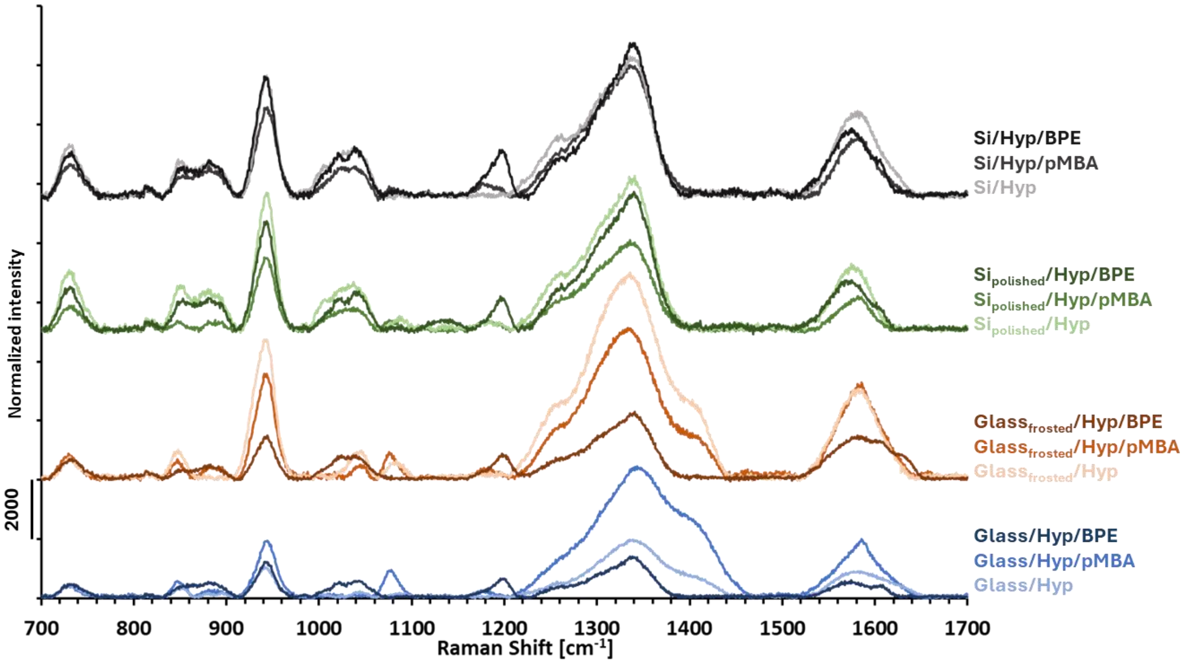

Figure S21. SERS spectra of clean substrates, pMBA, and BPE acquired for substrates made with AuNPs synthesized with L-(4)-hydroxyproline.

Table S4. The absorbance at the LSPR maximum of AuNPs utilized for the study. The absorbance is an average of three samples. The path length of the cuvette in which the measurement was taken was 1 cm. The results are based on our previous work [1].

AuNPs synthesized with	λ_{max} [nm]	Absorbance	SD
**Phe**	524	0.99	0.01
**Val**	522	0.83	0.01
**Hyp**	616	0.64	0.11
**Ser**	534	0.71	0.07
**Lys**	554	0.61	0.07

References

[1] Figat, A.M.; Bartosewicz, B.; Liszewska, M.; Budner, B.; Norek, M.; Jankiewicz, B.J. α-Amino Acids as Reducing and Capping Agents in Gold Nanoparticles Synthesis Using the Turkevich Method. *Langmuir* **2023**, *39*, 8646-8657. doi:10.1021/acs.langmuir.3c00507.

Table S5. The parameters of Raman measurements of SERS substrates.

Platform	Glass		Glass$_{frosted}$		Si$_{polished}$		Si	
	Acquisition time [s]; Accumulations	Percent of laser power [%]	Acquisition time [s]; Accumulations	Percent of laser power [%]	Acquisition time [s]; Accumulations	Percent of laser power [%]	Acquisition time [s]; Accumulations	Percent of laser power [%]
**Phe**	1; 1	1	1; 1	1	1; 1	1	1; 1	1
**Val**	1; 1	1	1; 1	1	1; 1	1	1; 1	1
**Hyp**	1; 1	1	1; 1	1	1; 1	1	1; 1	1
**Ser**	1; 1	0.0001	1; 1	0.0001	1; 1	1	1; 1	1
**Lys**	-	-	1; 1	1	-	-	-	-

Table S6. The parameters of SERS measurements of pMBA on various SERS substrates.

Platform	Glass		Glass$_{frosted}$		Si$_{polished}$		Si	
	Acquisition time [s]; Accumulations	Percent of laser power [%]	Acquisition time [s]; Accumulations	Percent of laser power [%]	Acquisition time [s]; Accumulations	Percent of laser power [%]	Acquisition time [s]; Accumulations	Percent of laser power [%]
**Phe**	1; 20	5	1; 20	5	1; 1	1	1; 1	1
**Val**	1; 1	1	1; 1	1	1; 1	1	1; 1	1
**Hyp**	2; 1	1	1; 1	1	1; 1	1	1; 1	1
**Ser**	1; 1	0.5	1; 1	0.5	1; 5	1	1; 5	1
**Lys**	-	-	1; 1	1	-	-	-	-

Table S7. The parameters of SERS measurements of BPE on various SERS substrates.

Platform	Glass		Glass$_{frosted}$		Si$_{polished}$		Si	
	Acquisition time [s]; Accumulations	Percent of laser power [%]	Acquisition time [s]; Accumulations	Percent of laser power [%]	Acquisition time [s]; Accumulations	Percent of laser power [%]	Acquisition time [s]; Accumulations	Percent of laser power [%]
**Phe**	1; 1	1	1; 1	1	1; 1	1	1; 1	1
**Val**	1; 1	1	1; 1	1	1; 1	1	1; 1	1
**Hyp**	1; 1	0.1	1; 1	0.1	1; 1	1	1; 1	1
**Ser**	1; 1	0.1	1; 1	0.1	1; 1	1	1; 1	1
**Lys**	-	-	1; 1	1	-	-	-	-

Procedure for evaluation of the total enhancement factor (EF)

The following procedure was used to evaluate the total enhancement factor of the SERS spectra obtained from pMBA molecules adsorbed at the fabricated SERS substrates (and for comparison, at the test Pt plate). Using the general formula for enhancement factors given in Eq. 1 in this article, we use the following data:

I_{SERS} - peak intensity value at 1075 cm^{-1} measured on SERS substrate, taking into account measurement parameters such as laser power, measurement time, and accumulations

I_{Raman} - peak intensity value at 1075 cm^{-1} measured on the Pt plate, taking into account measurement parameters such as laser power, measurement time, and accumulations

N_{vol} / N_{surf} - value is 1, because we assume that there is a monolayer of pMBA molecules on the surface of the SERS substrate and Pt plate. The pMBA deposition procedure and the pMBA concentration were the same in both cases.

The calculations also included the chemical enhancement factor for the pMBA, which was estimated in the Supplementary Data of the article by Weyher et al. [Weyher, J.L. et al. Relationship between the Nano-Structure of GaN Surfaces and SERS Efficiency: Chasing Hot-Spots. *Appl. Surf. Sci.* **2019**, *466*, 554-561. doi:10.1016/j.apsusc.2018.10.076] and was estimated to be 23.6. The chemical enhancement factor for the BPE was calculated using the same methodology and was found to be 37.2. The total enhancement factor was calculated by multiplying the result of Eq. 1 by the chemical enhancement factor. The same method of calculating EF was used for BPE.
